# Optimizing the Precision of Case Fatality Ratio Estimates Under the Surveillance Pyramid Approach

**DOI:** 10.1093/aje/kwu213

**Published:** 2014-09-25

**Authors:** Camille Pelat, Neil M. Ferguson, Peter J. White, Carrie Reed, Lyn Finelli, Simon Cauchemez, Christophe Fraser

**Keywords:** case fatality ratio, emerging infectious diseases, influenza, pandemics, statistical planning, surveillance protocol

## Abstract

In the management of emerging infectious disease epidemics, precise and accurate estimation of severity indices, such as the probability of death after developing symptoms—the symptomatic case fatality ratio (sCFR)—is essential. Estimation of the sCFR may require merging data gathered through different surveillance systems and surveys. Since different surveillance strategies provide different levels of precision and accuracy, there is need for a theory to help investigators select the strategy that maximizes these properties. Here, we study the precision of sCFR estimators that combine data from several levels of the severity pyramid. We derive a formula for the standard error, which helps us find the estimator with the best precision given fixed resources. We further propose rules of thumb for guiding the choice of strategy: For example, should surveillance of a particular severity level be started? Which level should be preferred? We derive a formula for the optimal allocation of resources between chosen surveillance levels and provide a simple approximation that can be used in thinking more heuristically about planning surveillance. We illustrate these concepts with numerical examples corresponding to 3 influenza pandemic scenarios. Finally, we review the equally important issue of accuracy.

During outbreaks of emerging infectious diseases, the symptomatic case fatality ratio (sCFR)—the probability of death following the development of symptoms—is a critical summary statistic characterizing disease severity, along with the probability of hospitalization given symptoms and the probability of symptoms given infection (1, 2). Estimates of the sCFR influence the public health measures put in place to control an epidemic. Large-scale public health responses are expensive and socially disruptive; health authorities often face a difficult tradeoff between mitigation and the costs to society (1). Accurate and precise estimates of the sCFR are essential for decision-making at the time response plans are being drawn up and as the plans are periodically revised during the course of the epidemic.

The 2009 A/H1N1 influenza pandemic illustrated well the challenges involved in rapid severity assessment. It was clear from early in the outbreak (3) that the severity of the epidemic was substantially less than that of the 1918 A/H1N1 pandemic (for which the sCFR was approximately 0.02 (4)), yet it proved more difficult to determine whether severity was *intermediate* (sCFR ≈ 10^−3^) or *mild* (sCFR ≈ 10^−4^) (5–13). Robust, precise estimates establishing that the pandemic had mild severity (sCFR = 4.5 × 10^−4^, 95% credible interval: 2 × 10^−4^, 9 × 10^−4^) were published by July 2009 (9).

In the context of an epidemic of *mild* severity, sample sizes required to directly estimate the sCFR by recording fatalities occurring in a series of symptomatic cases may become prohibitively large. As a consequence, several authors have developed pyramidal approaches to estimating the sCFR as a product of conditional probabilities—for example, the probability of hospitalization given symptoms times the probability of death upon symptom-related hospitalization (5, 10, 14). Strong assumptions underpin pyramidal approaches, particularly the assumption that deceased cases progress through the entire pyramid, which can limit their validity. However, these approaches present so many practical advantages that they were extensively used during the 2009 pandemic, and they can sometimes be the only available alternative.

Despite this fact, there is still little understanding of the statistical properties of pyramidal sCFR estimators. The standard error (SE), in particular, indicates when such estimators are more precise than direct estimation in a series of symptomatic cases. More generally, different pyramidal estimators, combining different surveillance systems and surveys, have different levels of precision. There is need for a theory to help in selecting the most precise estimator. Once the estimator has been selected, it is also unclear what the optimal allocation of resources between the different surveillance levels should be: Would it be better to conduct more outbreak investigations or to increase hospital-based surveillance? In an emerging infectious disease outbreak, where resources are finite, a clear strategy with which to efficiently reduce uncertainty around sCFR estimates is essential for management and planning.

In this article, we study the precision of pyramidal approaches to sCFR estimation and examine how the choice of the best approach depends upon outbreak characteristics. We propose rules of thumb for finding the most precise estimator and for optimizing resource allocation between surveillance levels. The general concepts are illustrated using 3 influenza pandemic scenarios with different levels of severity. We also discuss the issue of accuracy, which we propose may be best assessed on a case-by-case basis.

## METHODS

**Figure 1.**
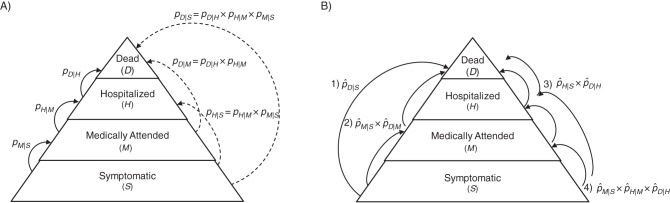
Classification of symptomatic cases of infectious disease into a severity pyramid comprising medical attention, hospitalization, and death (all related to symptomatic infection). A) Probabilities of progression to severity level *B* for persons on level *A*, *P*(*B*|*A*), denoted *p_B_*_|*A*_; for example, the probability of medical attention following symptoms is *p_M_* _|*S*_. Plain arrows indicate progression to the next level, and the dashed arrows indicate progression through intermediate level(s). B) Four estimators (numbered 1–4) of the symptomatic case fatality ratio, obtained either directly by following up symptomatic cases until death $\left({\hat{p}}_{D|S}\right)$ or by multiplying progression probabilities estimated from 2–3 surveillance levels. Outbreak investigations, household contact surveys, or symptomatic case series can be used to derive ${\hat{p}}_{M|S}$, ${\hat{p}}_{H|S}$, and ${\hat{p}}_{D|S}$; a surveillance system based on sentinel general practitioners can be used to derive ${\hat{p}}_{H|M}$ and ${\hat{p}}_{D|M}$; and a hospital-based surveillance system can be used to derive ${\hat{p}}_{D|H}$.

Pyramidal approaches to sCFR estimation combine data from several levels of the severity pyramid, making assumptions about the course of the disease and about health-care organization (5, 10, 14). For instance, in the severity pyramid of Figure 1, symptomatic cases (*S*) go through 2 severity levels before death (*D*): medical attention (*M*) and hospitalization (*H*). Assuming that all cases go through *M* before *H* and go through *H* before *D*, the sCFR can be written as a product of progression probabilities: sCFR = *P*(*D*|*H*) × *P*(*H*|*M*) × *P*(*M*|*S*) (see Web Appendix 1, available at http://aje.oxfordjournals.org/). A natural estimate of the sCFR is the product of the progression probability estimates. According to the available data at each severity level, different pyramidal estimators are possible, as long as they are based on reasonable assumptions. Figure 1 illustrates 4 estimators of the sCFR.

More generally, we are interested in comparing *K* pyramidal estimators, labeled *k* = 1 … *K*, with *N_k_* levels each. The sCFR in the *k*th estimation strategy is $\text{sCFR}=\prod_{i=1}^{N_{k}}\;p_{i,k}$, where *p_i_*_,*k*_ denotes the probability of progression from level *i* in strategy *k* to the next level and is estimated in a sample of *n_i_*_,*k*_ cases. The true value of the sCFR is independent of the estimation strategy, but the estimator $\left(s{\hat{\mathrm{CFR}}}_{k}=\prod_{i=1}^{N_{k}}{\hat{p}}_{i,k}\right)$ and its SE are not. Large SEs indicate low precision. A first-order approximation of the SE of pyramidal sCFR estimators is obtained with the delta method (15) (Web Appendix 2):

(1) $$\text{SE}(s{\hat{\mathrm{CFR}}}_{k})\approx \text{sCFR}\sqrt{\sum_{i=1}^{N_{k}}\frac{1}{n_{i,k}}\left(\frac{1}{\;p_{i,k}}-1\right)}.$$

The core of our paper is to study the precision of sCFR pyramidal estimators to find 1) the most precise estimation strategy and 2) the allocation of resources between a strategy's surveillance levels that maximizes precision.

### Optimizing resource allocation in a pyramidal approach

Sample sizes are constrained by the available number of cases at each severity level but also by the finite amount of (monetary) resources available for surveillance. We denote as *c_i_*_,*k*_ the cost of recruiting a case into the *i*th surveillance level of strategy *k*, so that $\sum_{i=1}^{N_{k}}c_{i,k}n_{i,k}$ is the cost of estimating sCFR with strategy *k* and sample sizes {*n_i_*_,*k*_}. We model resource constraints by assuming that there is a fixed budget *C*, and for each strategy *k* we ask, “What is the optimal allocation of resources to the different surveillance levels?” This involves finding sample sizes {*n_i_*_,*k*_} that minimize the SE subject to the constraint of the fixed budget: $\sum_{i=1}^{N_{k}}c_{i,k}n_{i,k}=C$. We show in Web Appendix 3 that this minimum is achieved for the following sample sizes:

(2) $$n_{i,k}^{*}\approx \frac{C}{c_{i,k}}\frac{\sqrt{c_{i,k}(1/\;p_{i,k}-1)}}{\sum_{\;j=1}^{N_{k}}\sqrt{c_{\;j,k}(1/\;p_{\;j,k}-1)}},\forall i=1,\ldots ,\;N_{k}.$$

A first observation from equation 2 is that the sample size $n_{i,k}^{*}$ increases as *p_i_*_,*k*_ decreases. This is obtained by studying the sign of $\partial (1/n_{i,k}^{*})/\partial p_{i,k}$: Being strictly positive on ]0;1[, it indicates that $n_{i,k}^{*}$ is a decreasing function of *p_i_*_,*k*_. Essentially, the rarer the events in each surveillance level, the more resources need to be assigned to it.

A second observation is that the optimal *proportion of resources* allocated to each surveillance level $\left(\rho_{i,k}^{*}=c_{i,k}\;\times n_{i,k}^{*}/C\right)$ is independent of the total budget *C*. A very simple approximation of this optimal proportion is obtained when the probabilities *p_i,k_* are much smaller than 1 and all of the costs *c_i_*_,*k*_ are equal (i.e., *c*_*i,k*_ = *c*, ∀*i*):

(3) $$\frac{n_{i,k}^{*}}{n}\approx \frac{1/\sqrt{\;p_{i,k}}}{\sum_{\;j=1}^{N_{k}}1/\sqrt{\;p_{\;j,k}}},\forall i=1,\ldots \;,\;N_{k},$$

where *n* is the total number of recruited cases (*n* = *C/c*). We suggest that this may serve as a useful heuristic for rapid surveillance setup: All else being equal, the proportion of cases that needs to be recruited at each surveillance level is proportional to the inverse square root of the probability of progression at that level.

We denote as $s{\hat{\mathrm{CFR}}}_{k}^{*}$ the estimator of strategy *k* when precision is optimum. Its SE is obtained by replacing {*n_i_*_,*k*_} with $\left\{n_{i,k}^{*}\right\}$ in equation 1:

(4) $$\text{SE}(s{\hat{\mathrm{CFR}}}_{k}^{*})\approx \frac{\text{sCFR}}{\sqrt{C}}\sum_{i=1}^{N_{k}}\sqrt{c_{i,k}\left(\frac{1}{\;p_{i,k}}-1\right)}.$$

Equation 4 can be inverted to calculate the budget necessary to reach a desired SE, σ, with strategy *k*:

(5) $$C\approx {\left[\frac{\text{sCFR}}{\sigma }\sum_{i=1}^{N_{k}}\sqrt{c_{i,k}\left(\frac{1}{p_{i,k}}-1\right)}\right]}^{2}$$

In some cases, it may be desirable to consider “recruitment needs” without reference to costs—for example, if it can be assumed that recruitment costs are equivalent between surveillance systems. Estimate precision is then a simple function of the total number of recruited cases *n*:

(6) $$\text{SE}(s{\hat{\mathrm{CFR}}}_{k}^{*})\approx \frac{\text{sCFR}}{\sqrt{n}}\sum_{i=1}^{N_{k}}\sqrt{\frac{1}{\;p_{i,k}}-1},$$

and the overall number of cases to recruit to reach a targeted SE, σ, is

(7) $$n\approx {\left[\frac{\text{sCFR}}{\sigma }\sum_{i=1}^{N_{k}}\sqrt{\frac{1}{p_{i,k}}-1}\right]}^{2}$$

In Web Appendix 3, we study the best allocation of resources 1) made available partway during an outbreak (Web Figure 1) and 2) given that some surveillance systems have fixed sample sizes (e.g., when routine surveillance data are reused for sCFR estimation).

### Choosing the most precise pyramidal estimator

While comparisons of SEs or necessary budgets can be carried out with equations 1, 4, and 5, we provide hereafter rules of thumb for rapid comparison of estimation strategies in the special case where all surveillance systems have equal recruitment costs and each estimator has a minimal SE thanks to appropriate allocation of resources.

We first study whether precision can be improved by adding a new surveillance level—that is, by splitting the progression probability *p_j_*_,*k*_ into 2 probabilities *p′* and *p*″ such that *p_j_*_,*k*_ = *p*′ × *p*″. In Web Appendix 4, we show that this split increases precision only if *p_j_*_,*k*_ < 1/9 (approximately 0.11) and if *p*′ and *p*″ satisfy

(8) $$\;\frac{\left(1-3\;p_{\;j,k}-\sqrt{1-10\;p_{\;j,k}+9{\;p_{\;j,k}}^{2}}\right)}{2}<\{p^{\prime },p^{\prime \prime }\}<\frac{\left(1-3\;p_{\;j,k}+\sqrt{1-10\;p_{\;j,k}+9{\;p_{\;j,k}}^{2}}\right)}{2}.$$

The smaller the value of *p_j_*_,*k*_, the wider this interval, so the less important this second condition is. For example, when *p_j_*_,*k*_ is the 2009 sCFR, all splits where *p*′ and *p*″ are picked between 0.00025 and 0.999 allow a gain in precision. This is illustrated in Web Figure 2.

We then study which pair *p*′ × *p*″ yields the most precise estimate. We show that the more similar *p*′ is to *p*″, the more precise the estimator, with maximum precision being reached when $p^{\prime }=p^{\prime \prime }=\sqrt{\;p_{j,k}}$ (see Web Appendix 4). These decision rules are summarized with a decision tree in Web Figure 3.

### Optimization in the presence of uncertainty

It is easy to calculate optimal sample sizes when all *p_i_*_,*k*_ are known, but in practice, of course, *p_i_*_,*k*_ are unknown. Yet informed guesses, denoted ${\tilde{p}}_{i,k}$, supported by the literature or by preliminary surveys, can be used to calculate the sCFR expected value, denoted $s\tilde{\mathrm{CFR}}=\prod_{i=1}^{N_{k}}{\tilde{p}}_{i,k}$, and the sample sizes, denoted ${\tilde{n}}_{i,k}$, that optimize estimator precision:

(9) $${\tilde{n}}_{i,k}\approx \frac{C}{c_{i,k}}\frac{\sqrt{c_{i,k}(1/{\tilde{p}}_{i,k}-1)}}{\sum_{\;j=1}^{N_{k}}\sqrt{c_{\;j,k}(1/{\tilde{p}}_{\;j,k}-1)}},\forall i=1,\;\ldots \;,\;N_{k}.$$

Through sensitivity analysis, developed in Web Appendix 5 (Web Figures 4–6), we examine how deviations of ${\tilde{p}}_{i,k}$ from the true value *p_i_*_,*k*_ affect the decided allocation of resources and, as a consequence, decrease estimator precision.

We also assess the robustness of the estimator choice to the initial uncertainty: Sensitivity techniques are used to construct 8 anticipation scenarios reflecting uncertainty about the severity of a particular outbreak as it starts. Robust performance ranking of sCFR estimators throughout all 8 scenarios is sought.

## RESULTS

The analytical results derived in the Methods section are illustrated below with numerical examples, using 3 influenza pandemic scenarios with different severity levels (Table 1):severe (“1918-like”; sCFR = 2%), intermediate (“1957-like”; sCFR = 0.2%), and mild (“2009-like”; sCFR =0.025%). We study the 4 estimators presented in Figure 1. Table 2 shows the key epidemiologic concepts illustrated in each example. For simplicity of illustration, we assume equal recruitment costs per case at all surveillance levels of all estimation strategies; consequently, the same budget allows for recruitment of the same number of cases. Other numerical examples, based on cost sets described Web Appendix 6 (Web Tables 1 and 2), are presented in Web Appendices 7 and 8 (Web Tables 3–6).

**Table 1.** Severity Parameters Used for Simulating 3 Influenza Pandemic Scenarios With Different Levels of Severity

Parameter	Description	Severity of Influenza Pandemic		
		Severe (1918-Like)	Intermediate (1957-Like)	Mild (2009-Like)
*p_D_*_|*S*_ = sCFR	Probability of death following symptoms	0.02040 (4)^a^	0.00020 (32)	0.00025 (14)
*p_M_*_|*S*_	Probability of medical attention following symptoms	0.4000	0.2000	0.3500
*p_H_*_|*M*_	Probability of hospitalization following medical attention	0.3500	0.1000	0.0157
*p_D_*_|*H*_	Probability of death following hospitalization	0.1457	0.1000	0.0455
*p_D_*_|*M*_ *= p_D_*_|*H*_ *× p_H_* _|*M*_	Probability of death following medical attention	0.0510	0.0100	0.0007
*p_H_* _|*S*_ *= p_H_*_|*M*_ *× p_M_*_|*S*_	Probability of hospitalization following symptoms	0.1400	0.0200	0.0055 (14)

Abbreviation: sCFR, symptomatic case fatality ratio.

^a^ For data found in the literature, source references are given in parentheses; the other figures are assumed.

**Table 2.** Key Concepts Illustrated in Numerical Examples Corresponding to 3 Influenza Pandemic Scenarios with Different Levels of Severity

Numerical Example	Methodological Concept(s)
Precision of surveillance strategies' pyramidal estimators	The optimal allocation of resources for any pyramidal estimator can be calculated with equation 2.
	The standard error of pyramidal sCFR estimators under optimal resource allocation can be calculated with equation 4.
	The precision of sCFR estimators increases when progression probabilities inferior to 0.11^a^ are split in two.
	The precision of sCFR estimators increases when multiplied progression probabilities are close to one another^a^.
Necessary budget	The minimal necessary budget for any pyramidal estimator to reach a desired precision can be calculated with equation 5.
	The minimal necessary budget for the single-level estimator increases dramatically as the sCFR decreases.
	Using pyramidal estimators allows a great precision gain when the sCFR is small.
Robustness to initial uncertainty	See “Optimization in the presence of uncertainty” in the Methods section of the text.

Abbreviation: sCFR, symptomatic case fatality ratio.

^a^ When recruitment costs at the concerned levels are equal.

### Precision of pyramidal estimators

**Figure 2.**
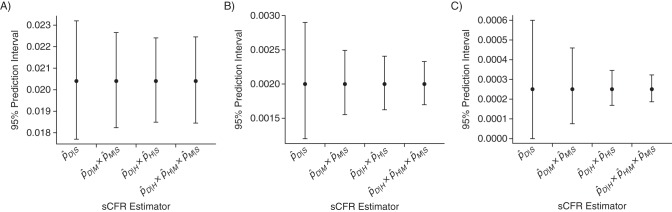
Ninety-five percent prediction intervals of symptomatic case fatality ratio (sCFR) estimators based on 10,000 recruited cases. Pyramidal sCFR estimators are obtained by multiplying estimates of *p_i_* _|*j*_, the probabilities of progressing to severity level *i* for cases in level *j*, which are based on 4 severity levels: symptomatic cases (*S*), medically attended cases (*M*), hospitalized cases (*H*), and dead cases (*D*). The 95% prediction intervals (T-shaped bars) are the intervals surrounding the true sCFRs (bullet points) in which future sCFR estimates will fall, with a probability of 95%, if the assumptions about progression probabilities are correct. Narrow prediction intervals indicate good precision. A) Severe (1918-like) influenza pandemic scenario; B) intermediate (1957-like) severity scenario; C) mild (2009-like) severity scenario. Maximal precision is ensured for each estimator through optimal resource allocation between surveillance levels. Recruitment costs are assumed to be equal at all surveillance levels of all estimation strategies.

**Figure 3.**
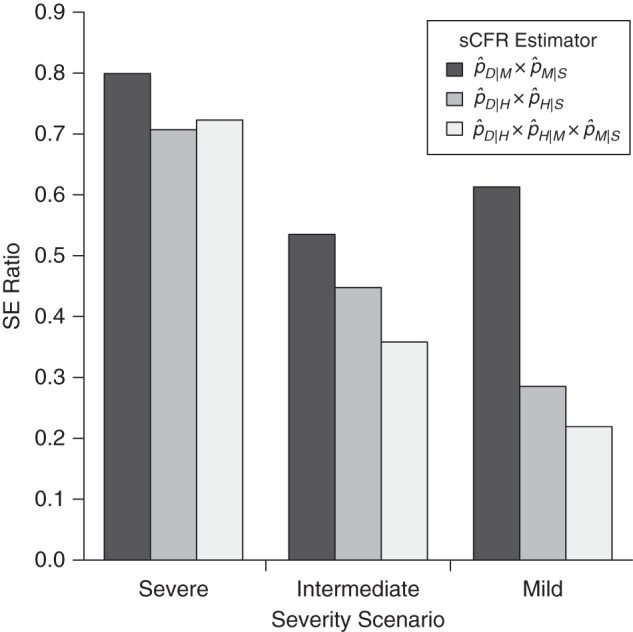
Relative precision of symptomatic case fatality ratio (sCFR) pyramidal estimators versus the single-level estimator $\left({\hat{p}}_{D|S}\right)$, given similar budgets. Relative precision is measured with the standard error (SE) ratio, obtained by dividing the minimal SE of each pyramidal estimator by that of the single-level estimator. Recruitment costs are assumed to be equal at all surveillance levels of all estimation strategies. It can be noted from equation 4 that the SE ratio of 2 estimators that have the same budget *C* is independent of *C*.

Table 3 presents the SE of the 4 sCFR estimators, obtained with equation 6, considering a budget allowing recruitment of 10,000 cases. The best possible precision is obtained for each estimator by appropriate allocation of resources between surveillance levels, calculated with equation 2. Figure 2 shows the 95% prediction intervals of the sCFR estimators, based on 10,000 recruited cases. Figure 3 presents SE ratios between the 3 pyramidal estimators and the single-level estimator. Precision usually increases with the number of surveillance levels in an estimation strategy, more so for small sCFRs. However, in our simulation, this was not systematic: In the severe scenario, a 2-level estimator $\left({\hat{p}}_{D|H}\times {\hat{p}}_{H|S}\right)$ was slightly more precise than the 3-level one $\left({\hat{p}}_{D|H}\times {\hat{p}}_{H|M}\;\times {\hat{p}}_{M|S}\right)$. This was predictable from the analytical results presented in the Methods section: The progression probability *p_H_* _|*S*_ is above 0.11 (*p_H_* _|*S*_ = 0.14), making it inefficient to further split it. The estimator ${\hat{p}}_{D|H}\times {\hat{p}}_{H|S}$ was always more precise than ${\hat{p}}_{D|M}\times {\hat{p}}_{M|S}$, with the same number of surveillance levels. This was also predictable: Estimators that multiply close progression probabilities are the most precise, and in all 3 scenarios *p_H_*_|*S*_ is closer to *p_D_*_|*H*_ than *p_M_*_|*S*_ is to *p_D_*_|*M*_ (see Table 1).

**Table 3.** Minimal Standard Errors of Symptomatic Case Fatality Ratio Estimators (Based on 10,000 Recruited Cases) When Recruitment Costs Are Equal at All Surveillance Levels^a^

Estimator	Level^b^	Event	Severity of Influenza Pandemic								
			Severe (1918-Like): sCFR = 2.04 × 10^−2^			Intermediate (1957-Like): sCFR = 2 × 10^−3^			Mild (2009-Like): sCFR = 2.5 × 10^−4^		
			Optimal Sample Size, no.	Expected No. of Events	SE (×10^−3^)	Optimal Sample Size, no.	Expected No. of Events	SE (×10^−4^)	Optimal Sample Size, no.	Expected No. of Events	SE (×10^−5^)
${\hat{p}}_{D|S}$	*S*	Death	10,000	204	1.41	10,000	20	4.47	10,000	3	15.81
${\hat{p}}_{D|M}\times {\hat{p}}_{M|S}$	*S*	Medical attention	2,211	885	1.13	1,674	335	2.39	352	123	9.69
	*M*	Death	7,789	397		8,326	83		9,648	7	
${\hat{p}}_{D|H}\times {\hat{p}}_{H|S}$	*S*	Hospitalization	5,058	708	1.00	7,000	140	2.00	7,459	41	4.51
	*H*	Death	4,942	720		3,000	300		2,541	116	
${\hat{p}}_{D|H}\times {\hat{p}}_{H|M}\times {\hat{p}}_{M|S}$	*S*	Medical attention	2,445	978	1.02	2,500	500	1.60	983	344	3.47
	*M*	Hospitalization	2,721	952		3,750	375		5,713	90	
	*H*	Death	4,834	704		3,750	375		3,304	150	

Abbreviations: sCFR, symptomatic case fatality ratio; SE, standard error.

^a^ The optimal sample size at each surveillance level is obtained with equation 2, ensuring the minimum SE for each estimator. Expected numbers of events are calculated as sample size × *p_i_*_|*j*_.

^b^ *H*, hospitalized cases; *M*, medically attended cases; *S*, symptomatic cases.

### Necessary budget

**Figure 4.**
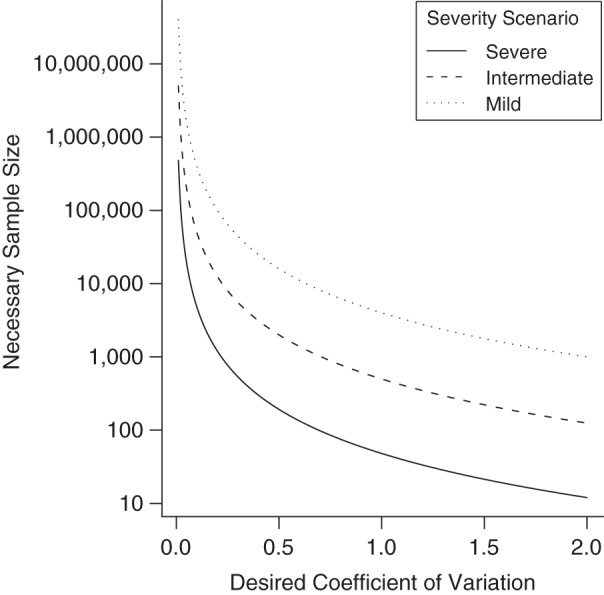
Sample size needed to reach different levels of precision with a single-level estimator of the symptomatic case fatality ratio. Low coefficients of variation indicate good precision. A single-level estimate may be obtained, for example, by counting fatalities in a symptomatic case series.

**Figure 5.**
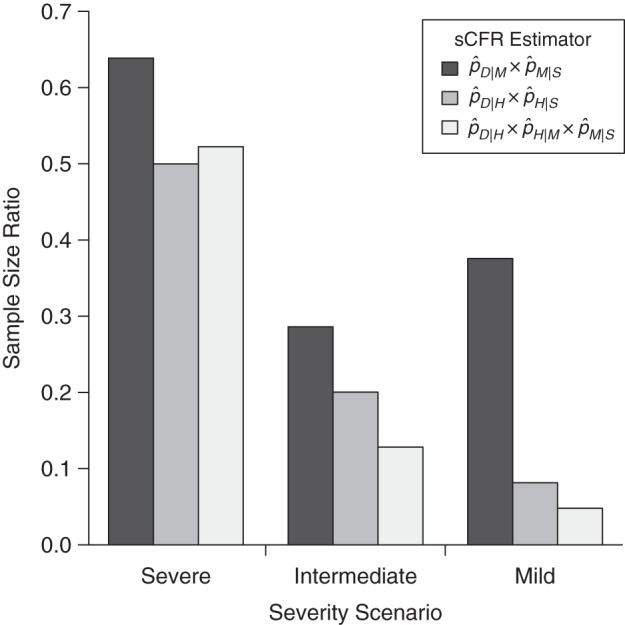
Relative necessary sample size of symptomatic case fatality ratio (sCFR) pyramidal estimators, in comparison with the single-level estimator ${\hat{p}}_{D|S}$. Sample size ratios divide the sample size needed to obtain a 0.5 coefficient of variation with pyramidal estimators by that of the single-level estimator. Maximal precision is ensured for each estimator through optimal resource allocation between surveillance levels. Recruitment costs are assumed to be equal at all surveillance levels of all strategies.

In this second illustration, we compare the necessary budgets, in terms of sample size, for all estimators to achieve the same level of precision. Again, we assume that the best possible precision is obtained for each estimator by appropriate allocation of resources. In Table 4, the number of cases needed to obtain a coefficient of variation (coefficient of variation = SE/sCFR) of 0.5 is calculated with equation 7. Figure 4 presents the necessary sample size of the single-level estimator for different targeted precisions. Figure 5 presents the relative sample sizes of the 3 pyramidal estimators. In short, choosing the optimal estimator substantially reduces recruitment efforts, particularly when the sCFR is low. This matters, because it is when the sCFR is low that such reduction is the most welcome, since necessary sample sizes for the single-level estimator are then prohibitive (Figure 4). For example, compared with the severe scenario, the size of a case series would need to be 10- and 83-fold larger for the intermediate and mild severity scenarios, respectively, for a single-level sCFR estimator to have a similar coefficient of variation. However, those differences are reduced when using the optimal pyramidal estimator in each scenario: One would then need 2.67- and 8-fold more cases for the intermediate and mild severity scenarios, respectively.

**Table 4.** Sample Sizes Necessary to Obtain a Coefficient of Variation of 0.5 for Symptomatic Case Fatality Ratio Estimators When Recruitment Costs Are Equal at All Surveillance Levels^a^

Estimator	Level^b^	Event	Severity of Influenza Pandemic								
			Severe (1918-Like): sCFR = 2.04 × 10^−2^			Intermediate (1957-Like): sCFR = 2 × 10^−3^			Mild (2009-Like): sCFR = 2.5 × 10^−4^		
			Optimal Sample Size, no.	Expected No. of Events	Cumulated Sample Size, no.	Optimal Sample Size, no.	Expected No. of Events	Cumulated Sample Size, no.	Optimal Sample Size, no.	Expected No. of Events	Cumulated Sample Size, no.
${\hat{p}}_{D|S}$	*S*	Death	192	4	192	1,996	4	1,996	15,996	4	15,996
${\hat{p}}_{D|M}\times {\hat{p}}_{M|S}$	*S*	Medical attention	27	11	123	96	19	572	211	74	6,011
	*M*	Death	96	5		476	5		5,800	4	
${\hat{p}}_{D|H}\times {\hat{p}}_{H|S}$	*S*	Hospitalization	49	7	96	280	6	400	972	5	1,303
	*H*	Death	47	7		120	12		331	15	
${\hat{p}}_{D|H}\times {\hat{p}}_{H|M}\times {\hat{p}}_{M|S}$	*S*	Medical attention	25	10	101	64	13	256	76	26	769
	*M*	Hospitalization	27	10		96	10		439	7	
	*H*	Death	49	7		96	10		254	12	

Abbreviation: sCFR, symptomatic case fatality ratio.

^a^ Corresponding standard errors are 1.02 × 10^−2^, 1.00 × 10^−3^, and 1.25 × 10^−4^ for the severe, intermediate, and mild scenarios, respectively. Optimal sample sizes are obtained by optimally allocating the total number of recruited cases (cumulated sample size) between surveillance levels using equation 2. Expected numbers of events are calculated as sample size × *p_i_* _|*j*_.

^b^ *H*, hospitalized cases; *M*, medically attended cases; *S*, symptomatic cases.

### Robustness to initial uncertainty

True optimization requires knowledge of the probabilities we want to estimate, which is obviously circular. To be useful, optimization needs to be robust to the use of approximate values available early during an emerging infectious disease outbreak. We analyze how uncertainty in the preliminary estimates of *p_i_*_,*k*_ may affect the optimal allocation of resources between surveillance levels. As an illustration, we study the estimator ${\hat{p}}_{D|H}\times {\hat{p}}_{H|S}$: We assume that the probability of death following hospitalization (*p_D_*_|*H*_) is known but the probability of hospitalization following symptoms (*p_H_*_|*S*_) is initially uncertain. We allow the preliminary estimate of *p_H_*_|*S*_ to vary between 0.0001 and 0.99. For each preliminary estimate of *p_H_*_|*S*_, we calculate the optimal allocation of resources between community-based surveillance systems and hospital-based surveillance systems. The more the preliminary estimate of *p_H_*_|*S*_ deviates from the true value, the less optimal is the resource allocation and the less precise is the sCFR estimator. However, we find numerically that estimator precision is quite robust even to errors of several orders of magnitude in the preliminary estimate of *p_H_*_|*S*_ (Web Figure 4).

For example, optimal precision for the mild scenario is obtained when 75% of cases are recruited in the community and 25% are recruited in hospitals. If the preliminary estimate of *p_H_*_|*S*_ were 0.0003 (instead of 0.0055), the initial “best guess” of optimal resource allocation would be 92% of cases recruited in the community and 8% of cases recruited in the hospital, yielding an SE 1.21-fold larger than optimal. Even with an initial estimate of *p_H_*_|*S*_ of 0.1, giving us a best-guess resource allocation ratio of 40%–60%, the SE is only 1.23-fold larger than optimal. Similar results are observed for the intermediate and severe severity scenarios and with the other 2-level estimator.

This implies that optimization of resources between surveillance levels can be based on an informed guess about progression probabilities, since only if this guess is wrong by several orders of magnitude will precision decrease substantially. This also means that gains in precision obtained by optimal resource allocation with a particular pyramidal estimator are much less substantial than gains in precision obtained from a good choice of the estimator.

**Figure 6.**
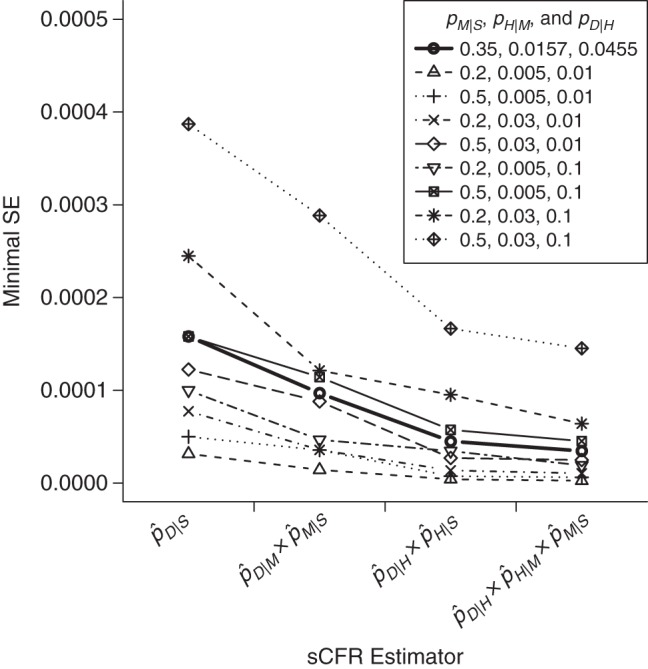
Expected minimal standard error (SE) of symptomatic case fatality ratio (sCFR) estimators in the presence of uncertainty around severity parameters, in a mild (2009-like) influenza pandemic scenario. A recruitment capacity of 10,000 cases is assumed. Parameters *p_M_*_|*S*_, *p_H_*_|*M*_, and *p_D_*_|*H*_ (the probabilities of medical attention upon symptoms, hospitalization upon medical attention, and death upon hospitalization, respectively) are assumed to be uncertain at the start of the pandemic. The true values of *p_M_*_|*S*_, *p_H_*_|*M*_, and *p_D_*_|*H*_ are 0.3500, 0.0157, and 0.0455, respectively, with uncertainty bounds of 0.2–0.5, 0.005–0.03, and 0.01–0.1, respectively. The minimal SE of each sCFR estimator is plotted for the true pandemic scenario (bold line) and for the 8 anticipation scenarios constructed by combining the uncertainty bounds. Those minimal SEs are obtained by optimally allocating the 10,000 recruited cases between surveillance levels and are calculated using equation 6.

Given this conclusion, we analyze whether the optimal estimator can be identified in the presence of uncertainty at the start of an outbreak. For each studied pandemic, we combine the initial uncertainty bounds of *p_M_*_|*S*_, *p_H_*_|*M*_, and *p_D_*_|*H*_ to obtain 8 anticipation scenarios. A consistent order of estimator precisions across all anticipation scenarios would strongly support the choice of the best one. In Figure 6, we present this analysis for the mild severity scenario assuming uncertainty ranges of 0.2–0.5, 0.005–0.03, and 0.01–0.1 for *p_M_*_|*S*_, *p_H_*_|*M*_, and *p_D_*_|*H*_, respectively (for the other scenarios, see Web Figures 5 and 6). For all 8 anticipation scenarios, the 3-level estimator is more precise than the others, with the single-level estimator always being the worst.

## DISCUSSION

Producing precise and accurate estimates of case fatality ratios early during an emerging infectious disease outbreak is important for public health decision-making. We have shown here that statistical planning can help investigators find an estimation strategy that minimizes costs without sacrificing precision. We focused on the sCFR as the case fatality ratio of most interest; however, the methods presented here can be used to optimize the precision of any severity estimate that decomposes into a product of probabilities, like the infectious case fatality ratio (making use of serology data) (16, 17).

We showed that the precision obtainable with a given budget depends greatly on the surveillance levels at which the estimation strategy is focused, and that pyramidal estimators with 3 surveillance levels can dramatically decrease the necessary budget in comparison with follow-up of a series of symptomatic cases. To a lesser extent, precision also depends on the allocation of resources between the surveillance levels of a given strategy.

As a result, we propose rules of thumb for finding the most precise estimator. First, precision generally increases with the number of surveillance levels, particularly when severity is low: Where feasible, surveillance levels should be further decomposed until the probability of progression to the next pyramid level is about 11% (at which point the gain in precision stops). Second, precision generally increases when the probabilities of progression multiplied together are similar. Finally, early identification of the optimal estimator is possible: Comparing the precision of all contemplated estimators over a plausible range of parameters guaranties a robust choice. Besides, as data are collected, severity parameters can be updated and surveillance strategies adapted.

Which estimator is ultimately preferred also depends on other criteria, such as the availability of data and the feasibility, timeliness, and quality of reporting, among others. Scaling up particular levels of a surveillance strategy might be difficult in real time; the limited precision gained by shuffling resources around surveillance levels must be contrasted with the associated inconvenience. Scaling up hospital-based surveillance or community case detection might be the most feasible option, the latter via outbreak investigation or Web or telephone cohorts. At the general practitioner level, one could imagine a system in which an aggregate number of cases is periodically reported, with outcome details on a proportion of these cases, and which could be scaled up or down depending on available data collection resources. When scaling up is done by enrolling more data providers, it is important to avoid introducing biases due to changes in the composition of the populations they serve (e.g., demographically). Finally, resource limitations might not be financial per se but might involve a limited number of staff; for example, suitably trained staff might be used to perform community contact tracing or might be sent to hospitals to extract data from patients’ case notes. This would be an example of reallocating resources between levels of a strategy.

Our study falls into the framework of *optimal designs,* which aim at estimating parameters without bias and with minimum variance (18); we have focused herein on the minimum variance issue. Few optimal design studies concern disease surveillance: Most have been simulation studies of surveillance protocols for animal diseases (19–23) and optimization studies of general practitioner recruitment for more precise estimation of influenza incidence in the community (24–27). More recently, Ejima et al. (28) used simulations to assess the delay before precise estimation of the case fatality ratio in various severity scenarios. We believe that the simple examples presented here provide pedagogical insight into how statistical planning may help improve the precision of sCFR estimates. However, these simple analyses had limitations.

First, we studied only precision (minimum variance), not accuracy (absence of bias). In particular, pyramidal estimators can be biased if some underlying assumptions are not satisfied—for example, in Figure 1, if *D* ⊂ *H* ⊂ *M* ⊂ *S* is not true. This is the case if a large proportion of deaths occur outside the hospital or if symptomatic cases are hospitalized without previous medical attention. In the 2009 A/H1N1 pandemic, the reported proportion of deaths taking place outside of hospitals was 17% in the United States (10), so it may be important to monitor death certification from primary care settings as well as hospitals, as was done in England (11), and use them in a comprehensive evidence synthesis approach: For example, Presanis et al. (10) estimated an overall sCFR by adding the sCFRs of hospitalized patients and nonhospitalized patients seen in general practice, both estimated with pyramidal estimators.

More generally, given how important severity estimates are early on in an outbreak and since, unfortunately, there is currently no real alternative to the pyramidal approach in a context of mild-to-intermediate sCFR, in the future it will be important to develop studies that can be conducted in parallel with pyramidal severity estimation to assess the validity and bias in the estimates. In particular, such studies should allow ascertainment of the likelihood of effectively going through each of the pyramids’ steps. For example, where a sample of symptomatic cases is used to estimate the probability of consulting a general practitioner upon development of symptoms, a subset can be used to also estimate the probability of hospitalization or death without a visit to a general practitioner. A random sample of death certificates can also be studied to identify influenza cases, reconstruct the patients’ health-care history, and determine the proportion that would not satisfy the pyramidal assumptions.

Biased pyramidal sCFR estimates can also result from reporting biases and inconsistent case ascertainment between surveillance levels. In this work, for simplicity's sake, we assumed that cases detected at any 1 level were representative and that the ascertainment of severity outcomes was complete. Reich et al. (29) discussed in detail the impact of reporting rates on estimation of the case fatality ratio. As an illustration of accuracy issues in pyramidal approaches, Presanis et al. (10) obtained 2 very different estimates of the 2009 A/H1N1 sCFR (0.048% and 0.007%) by using 2 pyramidal estimators, each one relying on a different set of reasonable assumptions. Each of these estimates had very tight credible intervals (i.e., good precision), and the 2 estimates were not consistent with each other.

This suggests a need for an analytical study of the accuracy of pyramidal estimators, which is outside the topic of the present paper. Garske et al. (5) described some of the mechanisms resulting in biases in sCFR pyramidal estimates during the 2009 pandemic. Accuracy will likely need to be ascertained on a case-by-case basis, and it may depend on organizational and cultural norms surrounding health-care utilization.

Second, we estimated progression probabilities with simple ratios so as to find an analytical solution to our optimization problem. These estimates can be significantly biased when the incidence of infection increases quickly and the delay between severity outcomes is long*.* Other authors have proposed estimation methods that account for right-truncation of severity events, which should be used in practice (5, 6, 29, 30).

Third, in Table 4 we compared strategies in terms of budget, yet the most economical strategy might not be the timeliest. For example, to estimate a 1957-like sCFR with a coefficient of variation of 0.5, one must “wait” either until 4 deaths are reported within a case series of 1,996 symptomatic cases or until 10 deaths are reported within a 3-level estimation strategy of 256 cases overall. Thus, while the latter strategy is the most economical (256 cases to recruit vs. 1,996), one has to wait for more fatal events to be reported. If time to death is highly variable, the delay before precise sCFR estimation may increase in pyramidal approaches, offsetting their economic superiority.

Fourth, we assumed fixed costs per recruitment at all surveillance levels. In practice, these costs can vary during an outbreak. This limitation could be addressed by using nonlinear cost functions. In addition, surveys and surveillance systems used in pyramidal approaches to sCFR estimation often are not specifically created for that purpose but are “reused,” avoiding the delays and costs of setting up ad hoc studies. We took this possibility into account, and in Web Appendix 3 we provide an analysis of optimal resource allocation in situations where some data sets have a fixed size; in the case of reusing precollected data sets, their cumulative cost can be set to 0.

Fifth, the probabilities of progression along the severity pyramid have to be stable through time in order for our method to be feasible. This might not be the case if, for example, the symptomatic population is strongly encouraged to consult a general practitioner midway through an outbreak, making *p_M_*_|*S*_, *p_H_*_|*M*_, and *p_D_*_|*M*_ suddenly change.

Finally, symptomatic cases may not all be homogeneous with regard to risk of disease and death. For instance, for influenza, while the risk of infection is typically higher among children, the risks of disease and death conditional on infection generally increase with age, except for the very young (31). Estimates of the sCFR can be stratified by risk group, provided that all surveillance levels used to generate estimates are similarly stratified, so that each stratum has its own independent pyramidal strategy. All of our considerations on precision and sample sizes then apply to each stratum.

To conclude, we have shown that statistical planning of surveillance strategies can help researchers achieve precise estimates of the sCFR with minimal costs through the judicious allocation of resources, even when those strategies are based on very approximate data early in an epidemic.

## Supplementary Material

Web Material
