# Supplementary material for: Optimizing the Precision of Case Fatality Ratio Estimates Under the Surveillance Pyramid Approach
Source: Am J Epidemiol. 2014 Sep 25;180(10):1036–46. doi: 10.1093/aje/kwu213 (PMC4240167; doi:10.1093/aje/kwu213)
Supplement: Web Material [file supp_180_10_1036__index.html]

Optimizing the Precision of Case Fatality Ratio Estimates Under the Surveillance Pyramid Approach — Optimizing the Precision of Case Fatality Ratio Estimates Under the Surveillance Pyramid Approach — Web Material 

# Optimizing the Precision of Case Fatality Ratio Estimates Under the Surveillance Pyramid Approach

## Web Material

Web Material

**Files in this Data Supplement:**

- Web Material - Pdf file
