# Supplementary material for: Optimizing the Precision of Case Fatality Ratio Estimates Under the Surveillance Pyramid Approach
Source: Am J Epidemiol. 2014 Sep 25;180(10):1036–46. doi: 10.1093/aje/kwu213 (PMC4240167; doi:10.1093/aje/kwu213)
Supplement: Web Material [file supp_kwu213_kwu213supp.pdf]

# “Optimizing the Precision of Case Fatality Ratio Estimates under the Surveillance Pyramid Approach” by Pelat et al.

**Web Appendix**

## Contents


Web Appendix 1. Derivation of sCFR pyramidal estimators ........ 2

Web Appendix 2. Derivation of the standard error of pyramidal estimators by the delta method........ 2

Web Appendix 3. Optimizing resource allocation in a pyramidal approach ........ 4

3.1 Demonstration of main-text equation 2........ 4

3.2 Optimal allocation of extra resources made available during an outbreak ........ 5

3.3 Optimal resource allocation given fixed surveillance systems ........ 9

Web Appendix 4. Rules of thumb for the comparison of estimators........ 11

4.1 When does a supplementary level improve precision?........ 11

4.2 Which intermediate level yields the most precise estimator? ........ 15

Web Appendix 5. Optimizing resource allocation in the presence of uncertainty........ 18

Web Appendix 6. Numerical examples with different sets of costs ........ 24

Web Appendix 7. Precision of pyramidal estimators when costs are different between surveillance levels........ 26

Web Appendix 8. Necessary budget when costs are different between surveillance levels........ 29

## Web Appendix 1. Derivation of sCFR pyramidal estimators

In the pyramid presented in main-text Figure 1, the assumption "medical attention always precedes hospitalization, which always precedes death" traduces mathematically as:

$$D \subset H \subset M \subset S .$$

Under such conditions, the symptomatic case fatality ratio (sCFR), $P(D|S)$, is equal to $P(D \cap H \cap M \mid S)$.

The latter decomposes into $P(D \mid H \cap M \cap S) \times P(H \mid \mathrm{M} \cap S) \times P(\mathrm{M} \mid S)$, thanks to Bayes' theorem, and finally simplifies as:

$$sCFR = P(D \mid H) \times P(H \mid \mathrm{M}) \times P(\mathrm{M} \mid S) .$$

## Web Appendix 2. Derivation of the standard error of pyramidal estimators by the delta method

The sCFR estimator provided by strategy $k$ is $sC\hat{F}R_k = \prod_{i=1}^{N_k} \hat{p}_{i,k}$, where $\hat{p}_{i,k}$ is obtained in a sample of $n_{i,k}$ cases at severity level $i$ by counting how many eventually reach level $i+1$ ($X_{i,k}$): $\hat{p}_{i,k} = X_{i,k} / n_{i,k}$ .

Let $\beta_k = \left[ p_{1,k}, p_{2,k}, ..., p_{N_k} \right]^T$ and $B_k = \left[ \hat{p}_{1,k}, \hat{p}_{2,k}, ..., \hat{p}_{N_k} \right]^T$. As all $\hat{p}_{i,k}$ are obtained on independent samples, we have the following variance-covariance matrix:

$$\mathrm{var}\left(B_k\right) = \begin{bmatrix} \mathrm{var}\left(\hat{p}_{1,k}\right) & 0 & ... & 0 \\ 0 & \mathrm{var}\left(\hat{p}_{2,k}\right) & ... & 0 \\ ... & ... & ... & ... \\ 0 & 0 & ... & \mathrm{var}\left(\hat{p}_{N_k,k}\right) \end{bmatrix}.$$

Let $h(.)$ be the product function: $h(\beta) = \prod_{i=1}^{N_k} p_{i,k} = sCFR$ and $h(B_k) = \prod_{i=1}^{N_k} \hat{p}_{i,k} = sC\hat{F}R_k$. The Delta method approximation gives $\operatorname{var}\left(h(B_k)\right) \approx \nabla h(\beta)^T . \operatorname{var}(B_k) . \nabla h(\beta)$, *i.e.*:

$$\operatorname{var}\left(sC\hat{F}R\right) \approx \begin{bmatrix} \dfrac{\partial \prod_{i=1}^{N_k} p_{i,k}}{\partial p_{1,k}} & \dfrac{\partial \prod_{i=1}^{N_k} p_{i,k}}{\partial p_{2,k}} & \cdots & \dfrac{\partial \prod_{i=1}^{N_k} p_{i,k}}{\partial p_{N_k,k}} \end{bmatrix} \begin{bmatrix} \operatorname{var}\left(\hat{p}_{1,k}\right) & 0 & \cdots & 0 \\ 0 & \operatorname{var}\left(\hat{p}_{2,k}\right) & \cdots & 0 \\ \cdots & \cdots & \cdots & \cdots \\ 0 & 0 & \cdots & \operatorname{var}\left(\hat{p}_{N_k,k}\right) \end{bmatrix} \begin{bmatrix} \dfrac{\partial \prod_{i=1}^{N_k} p_{i,k}}{\partial p_{1,k}} \\ \dfrac{\partial \prod_{i=1}^{N_k} p_{i,k}}{\partial p_{2,k}} \\ \cdots \\ \dfrac{\partial \prod_{i=1}^{N_k} p_{i,k}}{\partial p_{N_k,k}} \end{bmatrix}$$

$$\operatorname{var}\left(sC\hat{F}R\right) \approx \begin{bmatrix} \prod_{i=2}^{N_k} p_{i,k} & \prod_{\substack{i=1 \\ i\neq 2}}^{N_k} p_{i,k} & \cdots & \prod_{i=1}^{N_k-1} p_{i,k} \end{bmatrix} \begin{bmatrix} \operatorname{var}\left(\hat{p}_{1,k}\right) & 0 & \cdots & 0 \\ 0 & \operatorname{var}\left(\hat{p}_{2,k}\right) & \cdots & 0 \\ \cdots & \cdots & \cdots & \cdots \\ 0 & 0 & \cdots & \operatorname{var}\left(\hat{p}_{N_k,k}\right) \end{bmatrix} \begin{bmatrix} \prod_{i=2}^{N_k} p_{i,k} \\ \prod_{\substack{i=1 \\ i\neq 2}}^{N_k} p_{i,k} \\ \cdots \\ \prod_{i=1}^{N_k-1} p_{i,k} \end{bmatrix}$$

$$\operatorname{var}\left(sC\hat{F}R\right) \approx \left(\prod_{i=2}^{N_k} p_{i,k}\right)^2 \operatorname{var}\left(\hat{p}_{1,k}\right) + \left(\prod_{\substack{i=1 \\ i\neq 2}}^{N_k} p_{i,k}\right)^2 \operatorname{var}\left(\hat{p}_{2,k}\right) + \ldots + \left(\prod_{i=1}^{N_k-1} p_{i,k}\right)^2 \operatorname{var}\left(\hat{p}_{N_k,k}\right)$$

$$\operatorname{var}\left(sC\hat{F}R\right) \approx sCFR^2 \sum_{i=1}^{N_k} \frac{\operatorname{var}\left(\hat{p}_{i,k}\right)}{p_{i,k}{}^2}.$$

Assuming all $X_{i,k}$ follow binomial distributions, it comes that $\operatorname{var}\left(\hat{p}_{i,k}\right) = p_{i,k}\left(1 - p_{i,k}\right) / n_{i,k}$, so that $\operatorname{var}\left(sC\hat{F}R\right) \approx sCFR^2 \sum_{i=1}^{N_k} \frac{1}{n_{i,k}} \left(\frac{1}{p_{i,k}} - 1\right)$. The standard error (SE) being the square root of the variance, we obtain main-text equation 1: $\operatorname{SE}\left(sC\hat{F}R_k\right) \approx sCFR \sqrt{\sum_{i=1}^{N_k} \frac{1}{n_{i,k}} \left(\frac{1}{p_{i,k}} - 1\right)}$.

## Web Appendix 3. Optimizing resource allocation in a pyramidal approach

We thereafter provide the demonstration for main-text equation 2 that gives the optimal allocation of resources between the surveillance levels of a pyramidal approach to sCFR estimation in the general case. Then we make derivations in two special cases.

### 3.1 Demonstration of main-text equation 2

Main text equation 2 stipulates that, with a fixed surveillance budget C, the minimum SE of a sCFR estimator is achieved for the following sample sizes:

$$n_{i,k}^{*} \approx \frac{C}{c_{i,k}} \frac{\sqrt{c_{i,k}\left(\frac{1}{p_{i,k}}-1\right)}}{\sum_{j=1}^{N_k}\sqrt{c_{j,k}\left(\frac{1}{p_{j,k}}-1\right)}}, \forall i = 1,..,N_k .$$

<u>*Demonstration*</u>:

We seek $\theta_k^{\ *} = \left[n_{1,k}^{*}, n_{2,k}^{*}, ..., n_{N_k,k}^{*}\right]^T$ that minimizes $\mathrm{SE}\left(sC\hat{F}R_k\right)$ under the fixed budget constraint $\sum_{i=1}^{N_k} c_{i,k} n_{i,k} = C$. To that effect, we solve the following system of $N_k$ equations:

$$\begin{cases} \dfrac{\partial \mathrm{SE}^2\left(sC\hat{F}R_k\right)}{\partial n_{i,k}} = 0, \ \forall i = 1..N_k - 1 \\ \sum_{i=1}^{N_k} c_{i,k} n_{i,k} = C \end{cases} \quad (1)$$

where $\mathrm{SE}^2\left(sC\hat{F}R_k\right) \approx sCFR^2 \left( \left[\sum_{i=1}^{N_k-1} \frac{1}{n_{i,k}}\left(\frac{1}{p_{i,k}}-1\right)\right] + \frac{c_{N_k,k}}{C - \sum_{i=1}^{N_k-1} n_{i,k} c_{i,k}}\left(\frac{1}{p_{N_k,k}}-1\right) \right)$ is obtained by replacing $n_{N_k}$ with $\frac{1}{c_{N_k,k}}\left(C - \sum_{i=1}^{N_k-1} c_{i,k} n_{i,k}\right)$ in main-text equation 1. System (1) reduces to the following linear system:

$$\begin{cases} n_{i,k}\left(\sqrt{\dfrac{c_{N_k,k}c_{i,k}\left(\dfrac{1}{p_{N_k,k}}-1\right)}{\dfrac{1}{p_{i,k}}-1}}+c_{i,k}\right)+\sum_{\substack{j=1\\ j\neq i}}^{N_k}c_{j,k}n_{j,k}\approx C,\quad i=1..N_k-1 \\ \sum_{j=1}^{N_k}c_{j,k}n_{j,k}=C \end{cases} \tag{2}$$

Solving it by Gaussian elimination, we obtain the local extremum $\theta^*$ with all $n^*_{i,k}$ satisfying main-text equation 2.

It can further been proved, by considering the values of $\dfrac{\partial \mathrm{SE}^2\left(sC\hat{F}R_k\right)}{\partial n_{i,k}}$ at limits $n_i \to 0$ and $n_i \to C/c_{i,k}$ that $\theta^*$ is the global minimum of $\mathrm{SE}\left(sC\hat{F}R_k\right)$.

## 3.2 Optimal allocation of extra resources made available during an outbreak

An interesting case is when extra resources (*C'*) are made available to enhance surveillance part way through an outbreak. We study thereafter which surveillance systems to enhance to best improve precision. To that effect, we consider a two-level estimation strategy, with $n_1^0$ and $n_2^0$ the initial (and $n_1'$ and $n_2'$ the additional) numbers of cases collected at severity level 1 and 2, respectively. We seek $n_1'$ and $n_2'$ that minimize $\mathrm{SE}\left(sC\hat{F}R\right)$ under the resource constraint $C' = c_1n_1' + c_2n_2'$. Let $C = C' + c_1n_1^0 + c_2n_2^0$. We obtain the following solutions, which can be separated in 3 cases:

- if $n_1^0 \geq \frac{C}{c_1}\frac{\sqrt{c_1\left(\frac{1}{p_1}-1\right)}}{\sum_{j=1}^{2}\sqrt{c_j\left(\frac{1}{p_j}-1\right)}}$, $\mathrm{SE}\left(sC\hat{F}R\right)$ is minimum for $n'_1 = 0$ and $n'_2 = \frac{C'}{c_2}$. In other words, if the size of sample 1 size is above optimality given all available resources, the best thing to do is to focus on recruiting for sample 2.
- if $n_2^0 \geq \frac{C}{c_2}\frac{\sqrt{c_2\left(\frac{1}{p_2}-1\right)}}{\sum_{j=1}^{2}\sqrt{c_j\left(\frac{1}{p_j}-1\right)}}$, $\mathrm{SE}\left(sC\hat{F}R\right)$ is minimum for $n'_1 = \frac{C'}{c_1}$ and $n'_2 = 0$. In other words, if the size of sample 2 is above optimality given all available resources, the best thing to do is to focus on recruiting for sample 2.
- otherwise, $\mathrm{SE}\left(sC\hat{F}R\right)$ is minimum for $\left[n'_1\ ; n'_2\ \right]^{\mathrm{T}} = \left[n'^{*}_1 ; n'^{*}_2\right]^{\mathrm{T}}$ with

$$n'^{*}_i = \frac{C}{c_i}\frac{\sqrt{c_i\left(\frac{1}{p_i}-1\right)}}{\sum_{j=1}^{2}\sqrt{c_j\left(\frac{1}{p_j}-1\right)}} - n_i^0, i = 1,2. \quad (3)$$

In other words, in that last case, the additional resources are best used when split so as to reach optimal allocation of the total available resources (the ones invested so far plus the additional ones). Note that if $\left[n_1^0, n_2^0\right]$ are already optimally allocated, the optimal allocation for the additional resources *C'* is simply $n'^{*}_i \approx \frac{C'}{c_i}\frac{\sqrt{c_i\left(\frac{1}{p_i}-1\right)}}{\sum_{j=1}^{2}\sqrt{c_j\left(\frac{1}{p_j}-1\right)}}$.

**Numerical illustration: how to get the best out of extra resources**

Let us consider the estimator $\hat{p}_{D|H} \times \hat{p}_{H|S}$ and assume that a community survey has already recorded 100 confirmed cases for which hospitalization status is known, and that hospital-based surveillance is just starting to report cases. From this starting point, we study the gain in precision for each additional case recruited in the community or in hospital. Web Figure 1 shows the standard error as a function of the number of additional cases recruited in the community (*x*-axis) and in the hospital (*y*-axis). The plain lines show the optimal recruitment strategy. In the mild severity scenario, this consists of recruiting only hospitalized cases until 34 have been recruited and, from then on, to recruit three cases in the community (with known hospitalization status) for each case recruited in hospital (with known mortality outcome). Overall, the optimal resource allocation is 75% of cases being recruited at the community level and 25% at the hospital level (see main-text Table 3). In the severe pandemic scenario, the optimal recruitment strategy (having already detected 100 cases in the community) is to recruit only hospitalized cases until 97 of them have been accumulated and, from then on, recruit approximately equal numbers of cases in the community and hospitals.

**Web Figure 1**. Optimal recruitment strategy of additional cases, in a severe, intermediate and mild-severity influenza pandemic.

We consider a two-level pyramidal estimator based on community and hospital surveillance ( $\hat{p}_{D|H} \times \hat{p}_{H|S}$ ), assuming that 100 symptomatic cases have already been recruited in the community, and that new resources are made available to recruit more cases, both in the hospital and the community. Each new recruitment — in the community (*x*-axis) or in hospital (*y*-axis) — reduces the standard error (SE) of the sCFR estimator, which varies from high (black) to low (light gray). The plain line shows the recruitment strategy that best reduces the standard error for each recruited case; *e.g.* in the 2009-like mild scenario, this consists in recruiting first in hospital until reached 34 hospitalized cases, then recruiting about 3 cases in the community for each reported hospitalized case. This is traduced graphically by the gray line intercepting the *y*-axis at *y* = 34 and having a slope of 1/3.

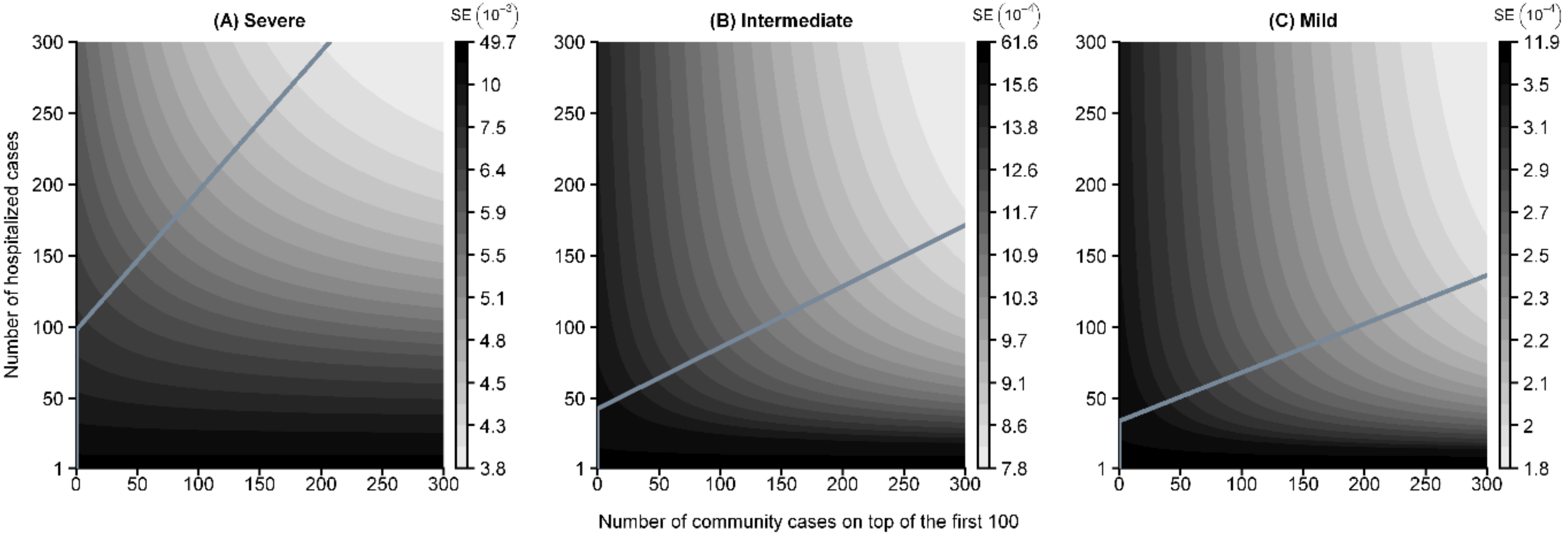

### 3.3 Optimal resource allocation given fixed surveillance systems

It may sometimes be the case that some surveillance systems (*e.g.* general practitioner sentinel networks) are already in place and have collected samples whose size cannot readily be changed during an emerging infectious disease outbreak. If those system use an amount $C'$ of the resources, the question is then how to optimally allocate the remaining resources ($C'' = C - C'$) to levels that are not covered by existing surveillance schemes. We show hereafter that this is obtained by optimizing the sample sizes of those ad-hoc surveillance systems/surveys given resources *C''* with a formula similar to main-text equation 2, regardless of the sample sizes of the surveillance systems already in place.

<u>*Demonstration*</u>:

Let assume that the sample sizes of levels 1 to *j*, $\left[n_{1,k}; n_{2,k}; ...; n_{j,k}\right]^{\mathrm{T}}$, are fixed. We seek $\theta^* = \left[n^*_{j+1,k}; n^*_{j+2,k}; ...; n^*_{N_k,k}\right]^{\mathrm{T}}$ that minimizes $\mathrm{SE}\left(s\hat{CFR}_k\right)$ while respecting the constraint $\sum_{i=j+1}^{N_k} n_{i,k} c_{i,k} = C''$. To that effect we solve analytically the following system of $N_k - j$ equations:

$$\begin{cases} \dfrac{\partial \mathrm{SE}^2\left(s\hat{CFR}_k\right)}{\partial n_{i,k}} = 0, \ \forall i = j+1..N_k - 1 \\ \sum_{i=j+1}^{N_k} c_{i,k} n_{i,k} = C'' \end{cases} \quad (4)$$

Where $\mathrm{SE}\left(s\hat{CFR}_k\right) \approx sCFR \sqrt{\sum_{i=1}^{N_k-1} \frac{1}{n_{i,k}}\left(\frac{1}{p_{i,k}} - 1\right) + \frac{c_{N_k,k}}{C'' - \sum_{i=j+1}^{N_k-1} n_{i,k} c_{i,k}}\left(\frac{1}{p_{N_k,k}} - 1\right)}$ is obtained by replacing $n_{N_k,k}$ with $\frac{1}{c_{N_k,k}}\left(C'' - \sum_{i=j+1}^{N_k-1} c_{i,k} n_{i,k}\right)$ in equation 1 of the main document. The system reduces to the following linear system:

$$\begin{cases} n_{i,k}\left(\sqrt{\dfrac{c_{N_k,k}c_{i,k}\left(\dfrac{1}{p_{N_k,k}}-1\right)}{\dfrac{1}{p_{i,k}}-1}}+c_{i,k}\right)+\sum_{\substack{l=j+1\\ l\neq i}}^{N_k} c_{l,k}n_{l,k}\approx C'', \quad i=j+1..N_k-1 \\ \sum_{l=j+1}^{N_k} c_{l,k}n_{l,k}=C'' \end{cases}$$

System (4) is similar to system (1) and can be solved in a similar manner. Thus, the sample sizes of studies $j+1$ to $N_k$ that minimize the standard error of the sCFR estimator are:

$$\theta^*=\left[n^*_{j+1,k};n^*_{j+2,k};...;n^*_{N_k,k}\right]^{\mathrm{T}} \text{ with } n^*_{i,k}\approx\frac{C''}{c_{i,k}}\frac{\sqrt{c_{i,k}\left(\dfrac{1}{p_{i,k}}-1\right)}}{\sum_{l=j+1}^{N_k}\sqrt{c_{l,k}\left(\dfrac{1}{p_{l,k}}-1\right)}} \qquad (5)$$

## Web Appendix 4. Rules of thumb for the comparison of estimators

The standard error ratio of two surveillance strategies with same budget *C* and optimal resource allocation between levels is independent of *C*:

$$\frac{\mathrm{SE}\left(sC\hat{F}R_k^*\right)}{\mathrm{SE}\left(sC\hat{F}R_l^*\right)} \approx \frac{\sum_{i=1}^{N_k}\sqrt{c_{i,k}\left(\frac{1}{p_{i,k}}-1\right)}}{\sum_{i=1}^{N_l}\sqrt{c_{i,l}\left(\frac{1}{p_{i,l}}-1\right)}} \tag{6}$$

We aim to find 1) when surveillance of a supplementary level improves precision and 2) how to best choose this level. <u>For mathematical tractability, we will suppose equal recruitment costs in all levels of all strategies.</u>

Consider an estimator *k* and an estimator *l*, built on estimator *k* by inserting an intermediate level. Specifically, the equality $p_{j,k} = p' \times p''$ is used to replace $\hat{p}_{j,k}$ by $\hat{p}' \times \hat{p}''$ in estimator *l*, all other progression probabilities being the same than in estimator *k*: $sC\hat{F}R_k = \prod_{i=1}^{N_k} \hat{p}_{i,k}$ and $sC\hat{F}R_l = \prod_{\substack{i=1 \\ i\neq j}}^{N_k} \hat{p}_{i,k} \times \hat{p}' \times \hat{p}''$.

### 4.1 When does a supplementary level improve precision?

<u>*Specifically*:</u> When does $\mathrm{SE}\left(sC\hat{F}R_l^*\right) < \mathrm{SE}\left(sC\hat{F}R_k^*\right)$ for the same budget *C*?

<u>*Mathematical derivations*:</u>

As we assume all recruitment cost per case (c.,.) equal, we use main-text equation 6 to define standard errors:

$$\mathrm{SE}\left(sC\hat{F}R_l^*\right) \approx \frac{sCFR}{\sqrt{n}}\sum_{i=1}^{N_l}\sqrt{\frac{1}{p_{i,l}}-1} \text{ and } \mathrm{SE}\left(sC\hat{F}R_k^*\right) \approx \frac{sCFR}{\sqrt{n}}\sum_{i=1}^{N_k}\sqrt{\frac{1}{p_{i,k}}-1}$$

Thus,

$$\mathrm{SE}\left(sC\hat{F}R_l^*\right) < \mathrm{SE}\left(sC\hat{F}R_k^*\right) \Leftrightarrow \sum_{i=1}^{N_l}\sqrt{\frac{1}{p_{i,l}}-1} < \sum_{i=1}^{N_k}\sqrt{\frac{1}{p_{i,k}}-1}$$
$$\Leftrightarrow \sum_{\substack{i=1\\ i\neq j}}^{N_k}\sqrt{\frac{1}{p_{i,k}}-1}+\sqrt{\frac{1}{p'}-1}+\sqrt{\frac{1}{p''}-1} < \sum_{\substack{i=1\\ i\neq j}}^{N_k}\sqrt{\frac{1}{p_{i,k}}-1}+\sqrt{\frac{1}{p_{j,k}}-1}$$

$$\Leftrightarrow \sqrt{\frac{1}{p'}-1}+\sqrt{\frac{1}{p''}-1} < \sqrt{\frac{1}{p_{j,k}}-1}$$

Taking each expression to the square, remembering that $p_{j,k} = p' \times p''$, and putting everything on the left hand-side, we obtain:

$$\mathrm{SE}\left(sC\hat{F}R_l^*\right) < \mathrm{SE}\left(sC\hat{F}R_k^*\right) \Leftrightarrow -\left[1-\frac{1}{p'}-\frac{p'}{p_{j,k}}+\frac{1}{p_{j,k}}\right]+2\sqrt{1-\frac{1}{p'}-\frac{p'}{p_{j,k}}+\frac{1}{p_{j,k}}} < 0 \qquad (7)$$

Letting $x = 1-\frac{1}{p'}-\frac{p'}{p_{j,k}}+\frac{1}{p_{j,k}}$, we obtain:

$$\begin{aligned}
&\mathrm{SE}\left(sC\hat{F}R_l^*\right) < \mathrm{SE}\left(sC\hat{F}R_k^*\right) \Leftrightarrow -x+2\sqrt{x} < 0\\
&\Leftrightarrow -\sqrt{x}+2 < 0\\
&\Leftrightarrow \sqrt{x} > 2\\
&\Leftrightarrow x > 4\\
&\Leftrightarrow \frac{1}{p_{j,k}}-\frac{1}{p'}-\frac{p'}{p_{j,k}}+1 > 4\\
&\Leftrightarrow \frac{1}{p_{j,k}}-\frac{1}{p'}-\frac{p'}{p_{j,k}}-3 > 0
\end{aligned}$$

Multiplying each side of the last inequality by *p'*, we obtain

$$\mathrm{SE}\left(sC\hat{F}R_l^*\right) < \mathrm{SE}\left(sC\hat{F}R_k^*\right) \Leftrightarrow -\frac{p'^2}{p_{j,k}}+\left(\frac{1}{p_{j,k}}+3\right)p'-1 > 0\,. \qquad (8)$$

Solving equation 8 in *p'*, it follows that:

1. If $p_{j.k} \in \left]\frac{1}{9};1\right[$, there is no real root for *p'*, and $\mathrm{SE}\left(sC\hat{F}R_l^*\right) > \mathrm{SE}\left(sC\hat{F}R_k^*\right)$ whatever $\{p', p''\}$ : this means that the splitting decreases precision.

2. If $p_{j.k} = 1/9$, $\mathrm{SE}\left(s\hat{CFR}_l^*\right) = \mathrm{SE}\left(s\hat{CFR}_k^*\right)$ if and only if $p' = p'' = \frac{1}{3}$, otherwise $\mathrm{SE}\left(s\hat{CFR}_l^*\right) > \mathrm{SE}\left(s\hat{CFR}_k^*\right)$ : the splitting decreases precision (or do not change it).

3. If $p_{j.k} \in \left]0;1/9\right[$, there are three cases:

    a. If $p' \in \left]\frac{1-3p_{j,k} - \sqrt{1-10p_{j,k} + 9p_{j,k}^2}}{2};\frac{1-3p_{j,k} + \sqrt{1-10p_{j,k} + 9p_{j,k}^2}}{2}\right[$ then $\mathrm{SE}\left(s\hat{CFR}_l^*\right) < \mathrm{SE}\left(s\hat{CFR}_k^*\right)$.

    b. If $p' \in \left]0;\frac{1-3p_{j,k} - \sqrt{1-10p_{j,k} + 9p_{j,k}^2}}{2}\right[ \cup \left]\frac{1-3p_{j,k} + \sqrt{1-10p_{j,k} + 9p_{j,k}^2}}{2};1\right[$ then $\mathrm{SE}\left(s\hat{CFR}_l^*\right) > \mathrm{SE}\left(s\hat{CFR}_k^*\right)$.

    c. If $p' = \frac{1-3p_{j,k} - \sqrt{1-10p_{j,k} + 9p_{j,k}^2}}{2}$ or $p' = \frac{1-3p_{j,k} + \sqrt{1-10p_{j,k} + 9p_{j,k}^2}}{2}$ then $\mathrm{SE}\left(s\hat{CFR}_l^*\right) = \mathrm{SE}\left(s\hat{CFR}_k^*\right)$.

This is illustrated in Web Figure 2, with $sCFR = p' \times p''$ : the standard error of the optimized two-level estimator is compared with that of the single-level estimator. The values $\{p', p''\}$ for which the single-level estimator (resp. the two-level estimator) is the most precise are highlighted in orange-red (resp. blue).

**Web Figure 2**. Ratio of the standard error of a two-level estimator of the symptomatic case fatality ratio ( $\hat{p}_{D|H} \times \hat{p}_{H|S}$ ) to a single-level estimator ( $\hat{p}_{D|S}$ ), for various $p_{H|S}$ and $p_{D|H}$ ( $p_{D|S} = p_{H|S} \times p_{D|H}$ ). The pairs $\{p_{H|S}, p_{D|H}\}$ for which the two-level estimator is more precise than the single-level one are in blue (darker blue for better precision). The ones for which the single-level estimator is more precise are in orange-red. Black plain lines: examples of $\{p_{H|S}, p_{D|H}\}$ pairs yielding a same sCFR: 0.00025 (as in 2009), or 1/9. Dashed line: $p_{H|S} = p_{D|H} = \sqrt{sCFR}$ .

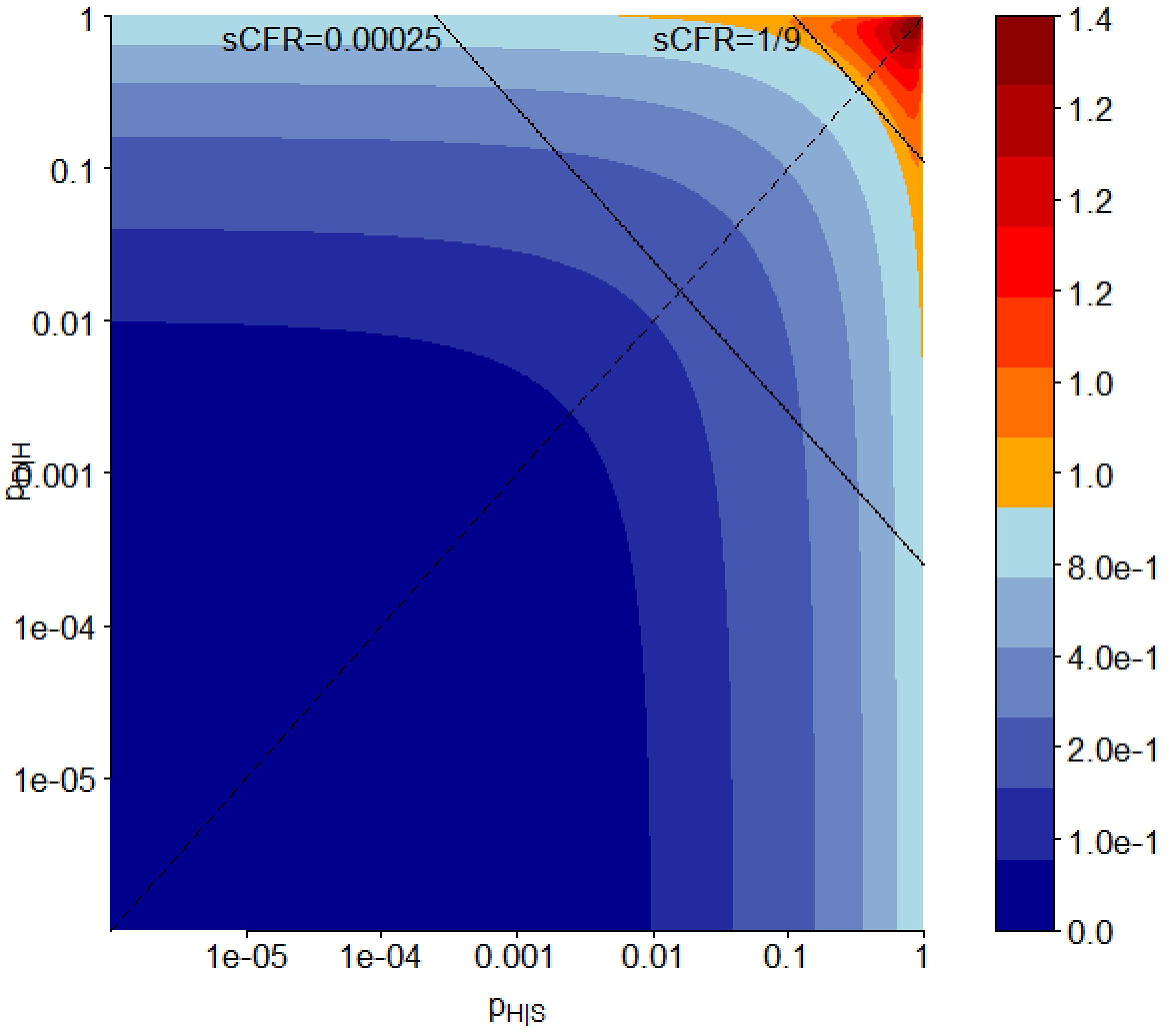

## 4.2 Which intermediate level yields the most precise estimator?

We seek $p'$ and $p''$ that minimize $\mathrm{SE}\left(sC\hat{F}R_l^*\right)$ under the constraint: $p_{i,k} = p' \times p''$.

We have: $\mathrm{SE}\left(sC\hat{F}R_l^*\right) \approx \frac{sCFR}{\sqrt{n}} \sum_{i=1}^{N_l} \sqrt{\frac{1}{p_{i,l}} - 1} \approx \frac{sCFR}{\sqrt{n}} \left[ \sum_{\substack{i=1 \\ i \neq j}}^{N_k} \sqrt{\frac{1}{p_{i,k}} - 1} + \sqrt{\frac{1}{p'} - 1} + \sqrt{\frac{1}{p_{j,k} / p'} - 1} \right]$.

Thus,

$$\frac{\partial \mathrm{SE}\left(sC\hat{F}R_l^*\right)}{\partial p'} \approx \frac{sCFR}{\sqrt{n}} \left[ \frac{1}{2\sqrt{\frac{1}{p'} - 1}} \left( -\frac{1}{p'^2} \right) + \frac{1}{2\sqrt{\frac{p'}{p_{j,k}} - 1}} \left( \frac{1}{p_{j,k}} \right) \right]$$

$$\frac{\partial \mathrm{SE}\left(sC\hat{F}R_l^*\right)}{\partial p'} \approx 0 \Leftrightarrow \frac{1}{2p'^2\sqrt{\frac{1}{p'} - 1}} \approx \frac{1}{2p_{j,k}\sqrt{\frac{p'}{p_{j,k}} - 1}}$$

$$\frac{\partial \mathrm{SE}\left(sC\hat{F}R_l^*\right)}{\partial p'} \approx 0 \Leftrightarrow \frac{1}{p'^4\left(\frac{1}{p'} - 1\right)} \approx \frac{1}{p_{j,k}{}^2\left(\frac{p'}{p_{j,k}} - 1\right)}$$

$$\frac{\partial \mathrm{SE}\left(sC\hat{F}R_l^*\right)}{\partial p'} \approx 0 \Leftrightarrow -p'^4 + p'^3 - p_{i,k}p' + p_{i,k}^2 \approx 0 \qquad (9)$$

Solving equation 9:

- If $p_{i,k} \geq 0.25$, polynomial (9) has 2 roots in $\Re$ : $p' = \sqrt{p_{i,k}}$ and $p' = -\sqrt{p_{i,k}}$. Only the first belongs to $]0;1[$; it is a local maximum for $\mathrm{SE}\left(sC\hat{F}R_l^*\right)$. Minima are obtained for $p' \to p_{i,k}$ and $p' \to 1$.

- If $p_{i,k} < 0.25$, there are 4 roots: $p' = \sqrt{p_{i,k}}$ , $p' = \frac{1-\sqrt{1-4p_{i,k}}}{2}$ , $p' = \frac{1+\sqrt{1-4p_{i,k}}}{2}$ and $p' = -\sqrt{p_{i,k}}$ , but only $p' = \sqrt{p_{i,k}}$ is a local minimum for $\mathrm{SE}\left(sC\hat{F}R_l^*\right)$ on $]0;1[$ .

We search under which condition $p' = \sqrt{p_{i,k}}$ is a global minimum *i.e.* which conditions make $\mathrm{SE}\left(sC\hat{F}R_l^*\right)\Big|_{p'=\sqrt{p_{j,,k}}} < \lim_{\substack{p'\to 1 \\ or \\ p'\to p_{i,k}}} \mathrm{SE}\left(sC\hat{F}R_l^*\right)$ true. It comes that $p' = \sqrt{p_{i,k}}$ is a global minimum **if and only if** $p_{i,k} < 1/9$ .

In conclusion, Web Figure 3 presents a decision tree that summarizes when to insert a supplementary surveillance level and how to best choose it.

**Web Figure 3**. Decision tree to find the most precise estimator between $s\hat{CFR}_k = \prod_{i=1}^{N_k} \hat{p}_{i,k}$ and $s\hat{CFR}_l = p' \times p'' \times \prod_{\substack{i=1 \\ i \neq j}}^{N_k} \hat{p}_{i,k}$ (resources optimally allocated within both). In the first estimation strategy, the progression probability $p_{j,k}$ is estimated in a single population; in the second, it is obtained by multiplying the estimates of progression probabilities $p'$ and $p''$.

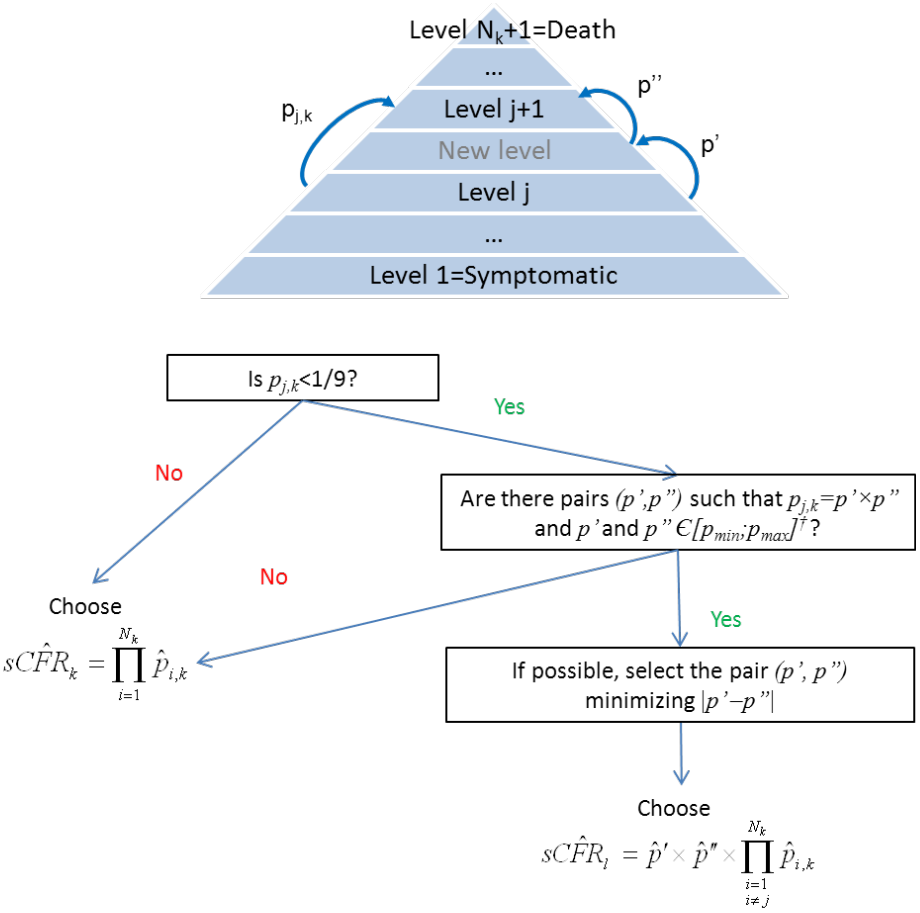


$$\dagger [p_{\min}; p_{\max}] = \left[ \frac{1-3p_{j,k} - \sqrt{1-10p_{j,k} + 9p_{j,k}^{\,2}}}{2} ; \frac{1-3p_{j,k} + \sqrt{1-10p_{j,k} + 9p_{j,k}^{\,2}}}{2} \right]$$

## Web Appendix 5. Optimizing resource allocation in the presence of uncertainty

We study the impact of initial uncertainty about severity parameters on resource allocation, and its consequence on the precision of sCFR estimators. Indeed, at the start of an outbreak, the probabilities $p_{i,k}$ are unknown and informed guesses, denoted $\tilde{p}_{i,k}$, supported by the literature or by preliminary surveys can be used to optimize resource allocation and calculate the expected precision of sCFR estimators. Thus, at the beginning of the outbreak the expected value of the sCFR is $s\tilde{CFR} = \prod_{i=1}^{N_k} \tilde{p}_{i,k}$ and the expected precision of estimator *k* with sample sizes $n_{i,k}$ is

$$\mathrm{SE}\left(s\hat{CFR}_k\right) \approx s\tilde{CFR}\sqrt{\sum_{i=1}^{N_k} \frac{1}{n_{i,k}}\left(\frac{1}{\tilde{p}_{i,k}} - 1\right)}. \qquad (10)$$

“Optimal” sample sizes (denoted $\tilde{n}_{i,k}$) based on the preliminary $\tilde{p}_{i,k}$ with budget *C* are

$$\tilde{n}_{i,k} \approx \frac{C}{c_{i,k}} \frac{\sqrt{c_{i,k}\left(\frac{1}{\tilde{p}_{i,k}} - 1\right)}}{\sum_{j=1}^{N_k}\sqrt{c_{j,k}\left(\frac{1}{\tilde{p}_{j,k}} - 1\right)}}, \forall i = 1,.., N_k, \qquad (11)$$

and the expected standard error with “optimal” sample sizes $\tilde{n}_{i,k}$ is

$$\frac{s\tilde{CFR}}{\sqrt{C}} \sum_{i=1}^{N_k} \sqrt{c_{i,k}\left(\frac{1}{\tilde{p}_{i,k}} - 1\right)}. \qquad (12)$$

However, given the true $p_{i,k}$, the standard error with sample sizes $\tilde{n}_{i,k}$ will be in reality:

$$\mathrm{SE}\left(s\hat{CFR}_k\right) \approx sCFR\sqrt{\sum_{i=1}^{N_k} \frac{1}{\tilde{n}_{i,k}}\left(\frac{1}{p_{i,k}} - 1\right)}. \qquad (13)$$

In Web Figure 4, we plot the 95% prediction interval (which is related to the standard error in equation 13) of the two-level estimators, when one probability of progression is uncertain at pandemic start. Prediction intervals increase as the preliminary estimate of the uncertain progression probability moves away from the true value, indicating lower precision. However, this increase is quite flat, indicating good robustness of the precision of sCFR estimators to initial uncertainty around severity parameters.

Web Figure 5 and Web Figure 6 reproduce main-text Figure 6 for the 1918- and 1957-like pandemic scenarios, respectively.

**Web Figure 4**. Precision of two estimators of the symptomatic case fatality ratio (sCFR) when optimal resource allocation is based on preliminary values.

We consider estimators $\hat{p}_{D|M} \times \hat{p}_{M|S}$ (column A) and $\hat{p}_{D|H} \times \hat{p}_{H|S}$ (column B), when $p_{M|S}$ and $p_{H|S}$, respectively, are uncertain. We make the preliminary value of $p_{M|S}$ (resp. $p_{H|S}$) vary in the top *x*-axis (log scale); to each preliminary value corresponds a calculated optimal size for the community symptomatic case sample (bottom *x*-axis) and a precision of the sCFR estimator (given by its 95% prediction interval, in *y*-axis). True sCFRs are indicated by horizontal plain lines. As preliminary $p_{M|S}$ (resp. $p_{H|S}$) is closer to its true value (dashed vertical line), the prediction interval narrows, indicating better precision.

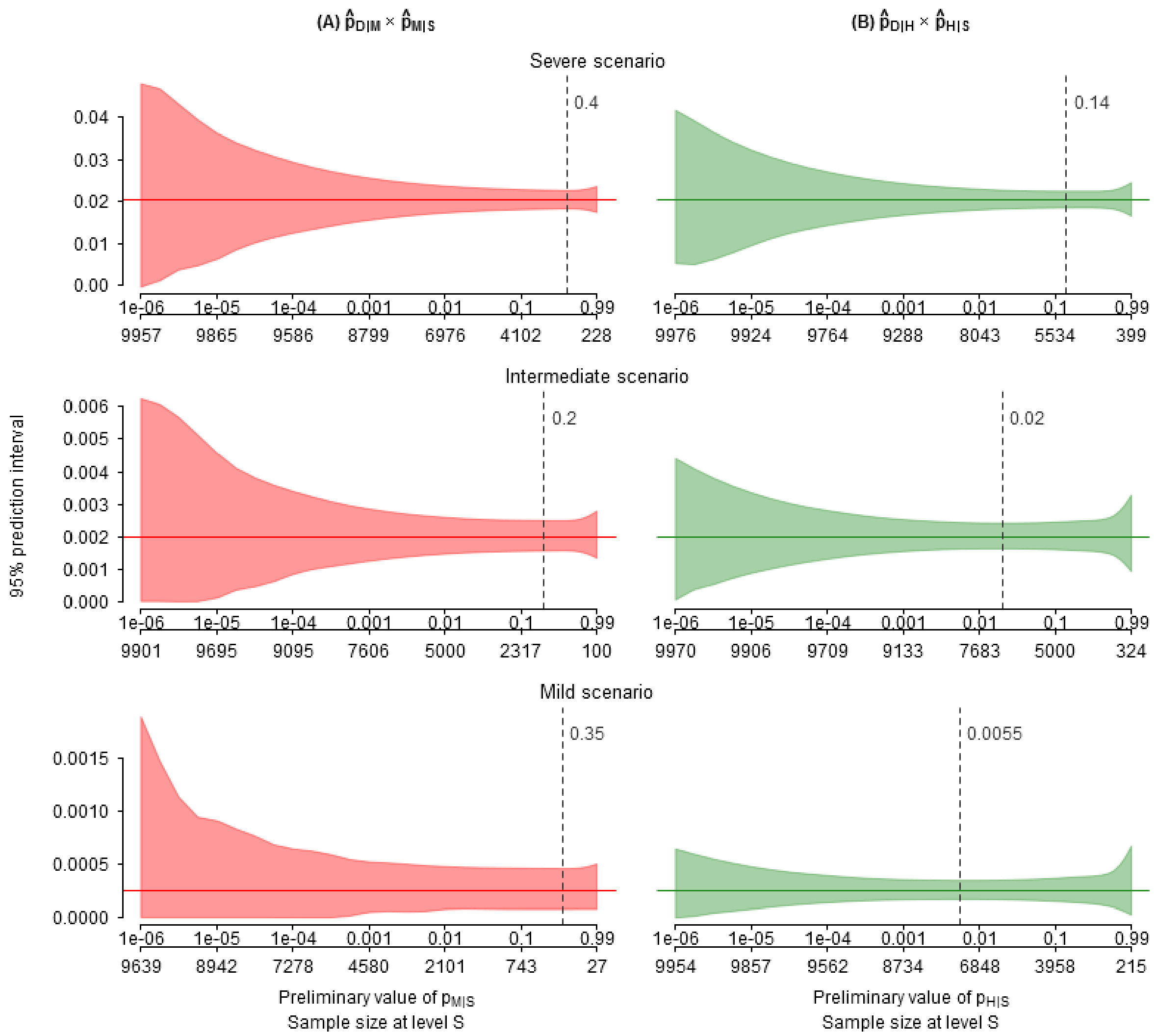


**Web Figure 5**. Expected standard error of symptomatic case fatality ratio (sCFR) estimators in the presence of uncertainty around severity parameters, in a severe 1918-like pandemic scenario.

A recruitment capacity of 10,000 cases is assumed. The parameters $p_{M|S}$, $p_{H|M}$ and $p_{D|H}$ (the probabilities of medical attention upon symptoms, hospitalization upon medical attention and death upon hospitalization, respectively) are supposed uncertain at pandemic start. The true values of $p_{M|S}$, $p_{H|M}$, and $p_{D|H}$ are 0.4, 0.35, and 0.1457, respectively, with uncertainty bounds 0.2–0.6, 0.15–0.35, and 0.05–0.3, respectively. The minimal standard error of each sCFR estimator is calculated for the true pandemic scenario and for the eight anticipation scenarios constructed by combining the uncertainty bounds (minimal standard errors are obtained by optimally allocating the 10,000 recruited cases between surveillance levels).

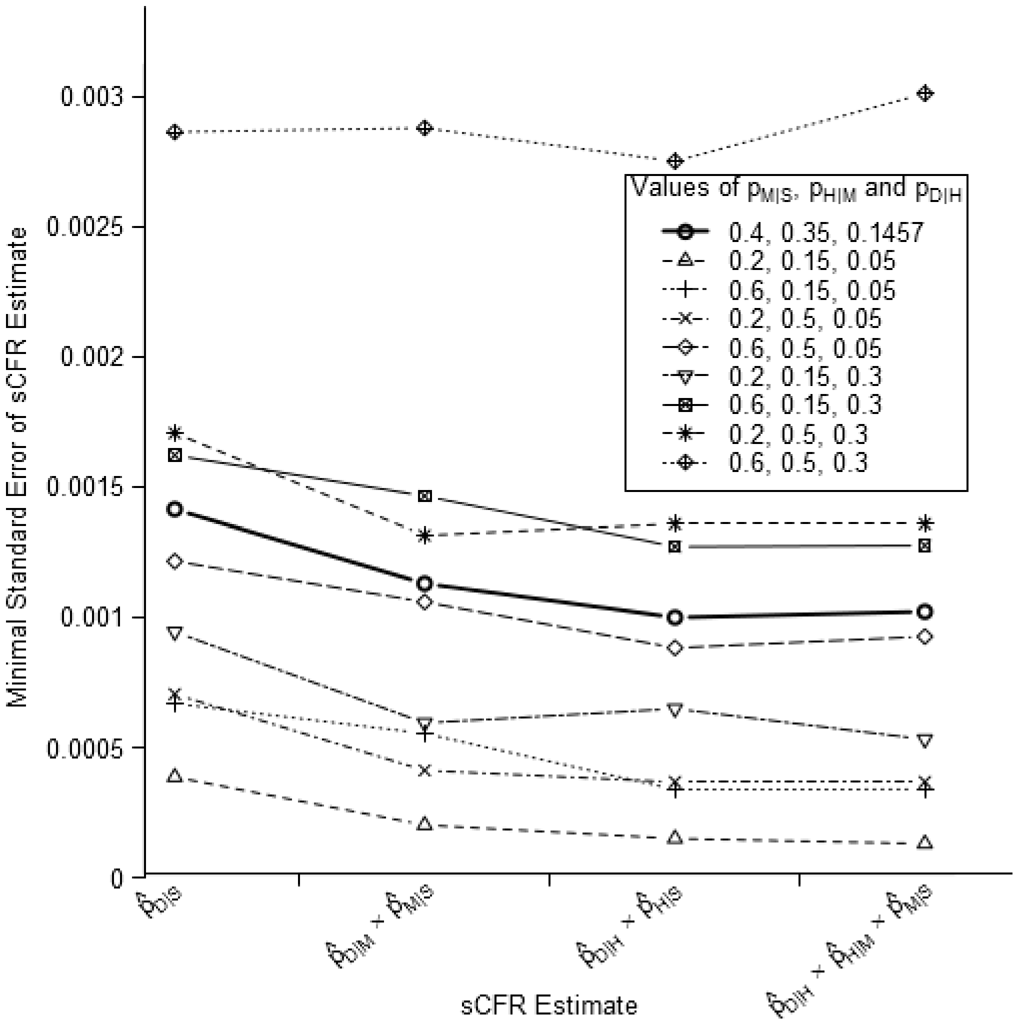


**Web Figure 6**. Expected standard error of symptomatic case fatality ratio (sCFR) estimators in the presence of uncertainty around severity parameters, in a severe 1918-like pandemic scenario.

A recruitment capacity of 10,000 cases is assumed. The parameters $p_{M|S}$, $p_{H|M}$ and $p_{D|H}$ (the probabilities of medical attention upon symptoms, hospitalization upon medical attention and death upon hospitalization, respectively) are supposed uncertain at pandemic start. The true values of $p_{M|S}$, $p_{H|M}$, and $p_{D|H}$ are 0.2, 0.1, and 0.1, respectively, with uncertainty bounds 0.05–0.2, 0.01–0.1, and 0.05–0.2, respectively. The minimal standard error of each sCFR estimator is calculated for the true pandemic scenario and for the eight anticipation scenarios constructed by combining the uncertainty bounds (minimal standard errors are obtained by optimally allocating the 10,000 recruited cases between surveillance levels).

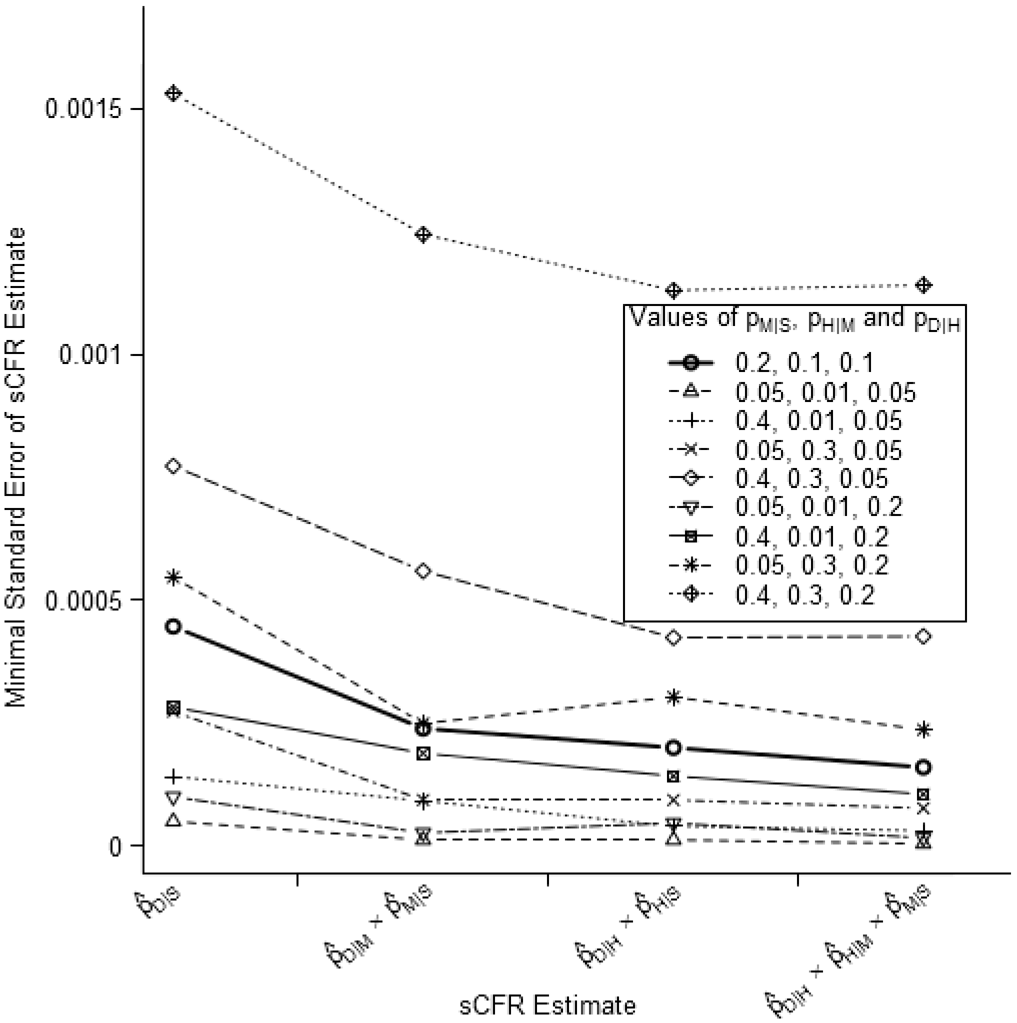

Minimal Standard Error of sCFR Estimate
0.0015
0.001
0.0005
0
Values of $p_{M|S}$, $p_{H|M}$ and $p_{D|H}$
0.2, 0.1, 0.1
0.05, 0.01, 0.05
0.4, 0.01, 0.05
0.05, 0.3, 0.05
0.4, 0.3, 0.05
0.05, 0.01, 0.2
0.4, 0.01, 0.2
0.05, 0.3, 0.2
0.4, 0.3, 0.2
$\hat{p}_{D|S}$
$\hat{p}_{D|M} \times \hat{p}_{M|S}$
$\hat{p}_{D|H} \times \hat{p}_{H|S}$
$\hat{p}_{D|H} \times \hat{p}_{H|M} \times \hat{p}_{M|S}$
sCFR Estimate

## Web Appendix 6. Numerical examples with different sets of costs

We consider thereafter two new sets of costs associated with the direct measure of progression probabilities ($p_{D|S}$, $p_{M|S}$, $p_{H|M}$, $p_{D|H}$, $p_{D|M}$, $p_{H|S}$) by ad-hoc surveillance systems or surveys. The progression probabilities remain the same as in main-text Table 1.

In the first set of costs (Web Table 1), we assume that, for each pandemic scenario, the highest cost ($5/case) is for recruiting symptomatic cases in the population and following them until definitive information on death (*i.e.* measuring directly $p_{D|S}$ on a case series). Following symptomatic cases until one obtains definitive information about a general practitioner (GP) visit ($p_{M|S}$) or hospitalization ($p_{H|S}$) costs $3/case. Obtaining from GPs the hospitalization status of symptomatic cases seen in consultation costs $2/case; obtaining death status costs $3/case. Finally, obtaining from hospitals information on death status only costs $1/hospitalized case.

Reversely, in the second set of costs (Web Table 2), we assume that obtaining any information on symptomatic cases from the population is cheap ($1 for obtaining death, hospitalization or GP visit status). Obtaining case data from GPs (hospitalization or death status) costs $2/case. Obtaining information on death from hospitals costs $3/case.

**Web Table 1**. First set of costs (in dollars ($) per recruited case) in different surveillance systems set for estimating different probabilities of progression.

| Scenario | $p_{D\|S}$ = *sCFR* | $p_{M\|S}$ | $p_{H\|M}$ | $p_{D\|H}$ | $p_{D\|M}$ | $p_{H\|S}$ |
|---|---|---|---|---|---|---|
| Severe (1918-like) | 5 | 3 | 2 | 1 | 3 | 3 |
| Intermediate (1957-like) | 5 | 3 | 2 | 1 | 3 | 3 |
| Mild (2009-like) | 5 | 3 | 2 | 1 | 3 | 3 |

**Web Table 2**. Second set of costs (in dollars ($) per recruited case) in different surveillance systems set for estimating different probabilities of progression.

| Scenario | $p_{D\|S}$ = *sCFR* | $p_{M\|S}$ | $p_{H\|M}$ | $p_{D\|H}$ | $p_{D\|M}$ | $p_{H\|S}$ |
|---|---|---|---|---|---|---|
| Severe (1918-like) | 1 | 1 | 2 | 3 | 2 | 1 |
| Intermediate (1957-like) | 1 | 1 | 2 | 3 | 2 | 1 |
| Mild (2009-like) | 1 | 1 | 2 | 3 | 2 | 1 |

## Web Appendix 7. Precision of pyramidal estimators when costs are different between surveillance levels

The standard error of the four sCFR estimators presented in main-text Figure 1 is calculated using the costs presented in Web Table 1 and Web Table 2, assuming a fixed budget of $10,000 for each estimation strategy. The optimal allocation of resources for each estimator is given by main-text equation 2. Standard errors are obtained with main-text equation 4.

Results

Using costs from Web Table 1, the precision gained by using pyramidal over single-level estimators is emphasized compared with the numerical example in the main text, which assumed equal costs at all surveillance levels (Web Table 3). Indeed, in Web Table 1, we stipulate that recruiting and following symptomatic cases from symptoms to death is expensive, which might well be the case in ad-hoc outbreak investigation survey. As a consequence, the three-level estimator is always the most precise, even in the 1918-like scenario (contrarily to what was observed when all costs were equal). In the 2009-like scenario, the standard error is reduced by as much as 87% compared with 78% when we used equal recruitment costs (see main-text Figure 3).

On the contrary, using costs from Web Table 2, the precision gained from using pyramidal estimators is decreased compared with the numerical example in the main text (Web Table 4). Indeed, in Web Table 2, recruiting and following symptomatic cases from the general population is cheap compared with general practitioner-based and hospital-based surveillance systems. In particular, in the 1918-like scenario, the single-level estimator is more precise than the two-level estimator based on general practitioners and the three-level estimator.

**Web Table 3**. Minimal Standard Error (SE) of sCFR Estimators, Based on a $10,000 Budget, When Recruitment Costs Are Those Provided in Web Table 1.[a]

| Estimator | Level | Event | Severe 1918-Like Pandemic sCFR = 2.04 × 10$^{-2}$ | | | Intermediate 1957-Like Pandemic sCFR = 2 × 10$^{-3}$ | | | Mild 2009-Like Pandemic sCFR = 2.5 × 10$^{-4}$ | | |
|---|---|---|---|---|---|---|---|---|---|---|---|
| | | | Optimal Sample Size, no. | Expected No. of Events | SE (×10$^{-3}$) | Optimal Sample Size, no. | Expected No. of Events | SE (×10$^{-4}$) | Optimal Sample Size, no. | Expected No. of Events | SE (×10$^{-5}$) |
| $\hat{p}_{D\|S}$ | S | Death | 2,000 | 41 | 3.16 | 2,000 | 4 | 9.99 | 2,000 | 0.5 | 35.35 |
| $\hat{p}_{D\|M} \times \hat{p}_{M\|S}$ | S | Medical attention | 737 | 295 | 1.96 | 558 | 112 | 4.14 | 117 | 41 | 16.79 |
| | M | Death | 2,596 | 132 | | 2,775 | 28 | | 3,216 | 2 | |
| $\hat{p}_{D\|H} \times \hat{p}_{H\|S}$ | S | Hospitalization | 2,131 | 298 | 1.37 | 2,672 | 53 | 3.02 | 2,786 | 15 | 6.97 |
| | H | Death | 3,606 | 525 | | 1,984 | 198 | | 1,643 | 75 | |
| $\hat{p}_{D\|H} \times \hat{p}_{H\|M} \times \hat{p}_{M\|S}$ | S | Medical attention | 1,093 | 437 | 1.32 | 1,078 | 216 | 2.14 | 434 | 152 | 4.53 |
| | M | Hospitalization | 1,489 | 521 | | 1,981 | 198 | | 3,087 | 48 | |
| | H | Death | 3,742 | 545 | | 2,802 | 280 | | 2,525 | 115 | |

[a] The optimal sample size at each surveillance level is obtained with main-text equation 2. Expected numbers of events are calculated as sample size × $p_{i|j}$.

sCFR: symptomatic case fatality ratio; S: symptomatic cases; M: medically attended cases; H: hospitalized cases.

**Web Table 4**. Minimal Standard Error (SE) of sCFR Estimators, Based on a $10,000 Budget, When Recruitment Costs Are Those Provided in Web Table 2[a]

| Estimator | Level | Event | Severe 1918-Like Pandemic sCFR = 2.04 × $10^{-2}$ | | | Intermediate 1957-Like Pandemic sCFR = 2 × $10^{-3}$ | | | Mild 2009-Like Pandemic sCFR = 2.5 × $10^{-4}$ | | |
|---|---|---|---|---|---|---|---|---|---|---|---|
| | | | Optimal Sample Size, no. | Expected No. of Events | SE (×$10^{-3}$) | Optimal Sample Size, no. | Expected No. of Events | SE (×$10^{-4}$) | Optimal Sample Size, no. | Expected No. of Events | SE (×$10^{-5}$) |
| $\hat{p}_{D\|S}$ | S | Death | 10,000 | 204 | 1.41 | 10,000 | 20 | 4.47 | 10,000 | 2 | 15.81 |
| $\hat{p}_{D\|M} \times \hat{p}_{M\|S}$ | S | Medical attention | 1,672 | 669 | 1.49 | 1,244 | 249 | 3.21 | 251 | 88 | 13.56 |
| | M | Death | 4,164 | 212 | | 4,378 | 44 | | 4,874 | 3 | |
| $\hat{p}_{D\|H} \times \hat{p}_{H\|S}$ | S | Hospitalization | 3,715 | 520 | 1.36 | 5,740 | 115 | 2.44 | 6,289 | 35 | 5.35 |
| | H | Death | 2,095 | 305 | | 1,420 | 142 | | 1,237 | 56 | |
| $\hat{p}_{D\|H} \times \hat{p}_{H\|M} \times \hat{p}_{M\|S}$ | S | Medical attention | 1,667 | 667 | 1.5 | 1,748 | 350 | 2.29 | 665 | 233 | 5.12 |
| | M | Hospitalization | 1,312 | 459 | | 1,854 | 185 | | 2,732 | 43 | |
| | H | Death | 1,903 | 277 | | 1,514 | 151 | | 1,290 | 59 | |

[a] The optimal sample size at each surveillance level is obtained with main-text equation 2. Expected numbers of events are calculated as sample size × $p_{i|j}$.

sCFR: symptomatic case fatality ratio; S: symptomatic cases; M: medically attended cases; H: hospitalized cases.

## Web Appendix 8. Necessary budget when costs are different between surveillance levels

We now use the sets of costs provided in Web Table 1 and Web Table 2 to calculate the necessary budget to obtain a predefined precision level. For example, we aim to obtain a coefficient of variation of 0.5, for each estimator in each pandemic scenario. The absolute results are presented below in Web Table 5 and Web Table 6.

In the 2009-like pandemic scenario, the necessary budget can be reduced by 98% (resp. 89%) by using the three-level estimator instead of the single-level one, when costs are those specified in Web Table 1 (resp. Web Table 2). It was 95% when all surveillance costs were equal.

In the 1918-like scenario using the three-level estimator allows reducing the necessary budget by 62% when costs are those specified in Web Table 1, but increases them by 12% when costs are those specified in Web Table 2. It was a 36% budget decrease when all surveillance costs were equal.

**Web Table 5**. Necessary Budget to Obtain a Coefficient of Variation of 0.5 for sCFR Estimators, When Recruitment Costs Are Those Provided in Web Table 1[a]

| Estimator | Level | Event | Severe 1918-Like Pandemic sCFR = $2.04 \times 10^{-2}$ | | | Intermediate 1957-Like Pandemic sCFR = $2 \times 10^{-3}$ | | | Mild 2009-Like Pandemic sCFR = $2.5 \times 10^{-4}$ | | |
|---|---|---|---|---|---|---|---|---|---|---|---|
| | | | Optimal Sample Size, no. | Expected No. of Events | Necessary Budget | Optimal Sample Size, no. | Expected No. of Events | Necessary Budget | Optimal Sample Size, no. | Expected No. of Events | Necessary Budget |
| $\hat{p}_{D\|S}$ | S | Death | 192 | 4 | 960 | 1,996 | 4 | 9,980 | 15,996 | 4 | 79,980 |
| $\hat{p}_{D\|M} \times \hat{p}_{M\|S}$ | S | Medical attention | 27 | 11 | 368 | 96 | 19 | 1,714 | 211 | 74 | 18,034 |
| | M | Death | 96 | 5 | | 476 | 5 | | 5,800 | 4 | |
| $\hat{p}_{D\|H} \times \hat{p}_{H\|S}$ | S | Hospitalization | 38 | 5 | 180 | 244 | 5 | 915 | 867 | 5 | 3,113 |
| | H | Death | 65 | 9 | | 181 | 18 | | 512 | 23 | |
| $\hat{p}_{D\|H} \times \hat{p}_{H\|M} \times \hat{p}_{M\|S}$ | S | Medical attention | 18 | 7 | 167 | 49 | 10 | 459 | 57 | 20 | 1,316 |
| | M | Hospitalization | 25 | 9 | | 91 | 9 | | 406 | 6 | |
| | H | Death | 63 | 9 | | 128 | 13 | | 332 | 15 | |

[a] Corresponding standard errors are $1.02 \times 10^{-2}$, $1.00 \times 10^{-3}$, and $1.25 \times 10^{-4}$, for the severe, intermediate and mild scenario, respectively. Optimal sample sizes are obtained by optimally allocating the total number of recruited cases (cumulated sample size) between surveillance levels using main-text equation 2. Expected numbers of events are calculated as sample size × $p_{i|j}$.

sCFR: symptomatic case fatality ratio; S: symptomatic cases; M: medically attended cases; H: hospitalized cases.

**Web Table 6**. Necessary Budget to Obtain a Coefficient of Variation of 0.5 for sCFR Estimators, When Recruitment Costs Are Those Provided in Web Table 2. [a]

| Estimator | Level | Event | Severe 1918-Like Pandemic sCFR = 2.04 × $10^{-2}$ | | | Intermediate 1957-Like Pandemic sCFR = 2 × $10^{-3}$ | | | Mild 2009-Like Pandemic sCFR = 2.5 × $10^{-4}$ | | |
|---|---|---|---|---|---|---|---|---|---|---|---|
| | | | Optimal Sample Size, no. | Expected No. of Events | Necessary Budget | Optimal Sample Size, no. | Expected No. of Events | Necessary Budget | Optimal Sample Size, no. | Expected No. of Events | Necessary Budget |
| $\hat{p}_{D\|S}$ | S | Death | 192 | 4 | 192 | 1,996 | 4 | 1,996 | 15,996 | 4 | 15,996 |
| $\hat{p}_{D\|M} \times \hat{p}_{M\|S}$ | S | Medical attention | 36 | 14 | 215 | 129 | 26 | 1,033 | 296 | 104 | 11,776 |
| | M | Death | 89 | 5 | | 452 | 5 | | 5,740 | 4 | |
| $\hat{p}_{D\|H} \times \hat{p}_{H\|S}$ | S | Hospitalization | 66 | 9 | 178 | 341 | 7 | 595 | 1,152 | 6 | 1,832 |
| | H | Death | 37 | 5 | | 84 | 8 | | 227 | 10 | |
| $\hat{p}_{D\|H} \times \hat{p}_{H\|M} \times \hat{p}_{M\|S}$ | S | Medical attention | 36 | 14 | 216 | 92 | 18 | 523 | 112 | 39 | 1,680 |
| | M | Hospitalization | 28 | 10 | | 97 | 10 | | 459 | 7 | |
| | H | Death | 41 | 6 | | 79 | 8 | | 217 | 10 | |

[a] Corresponding standard errors are 1.02 × $10^{-2}$, 1.00 × $10^{-3}$, and 1.25 × $10^{-4}$, for the severe, intermediate and mild scenario, respectively. Optimal sample sizes are obtained by optimally allocating the total number of recruited cases (cumulated sample size) between surveillance levels using main-text equation 2. Expected numbers of events are calculated as sample size × $p_{i|j}$.
